# Adverse event profiles of EGFR-TKI: network meta-analysis and disproportionality analysis of the FAERS database

**DOI:** 10.3389/fphar.2025.1519849

**Published:** 2025-03-11

**Authors:** Jing Shi, Xinya Liu, Mengjiao Gao, Jian Yu, Ting Chai, Yun Jiang, Jiawei Li, Yuanming Zhang, Li Wu

**Affiliations:** ^1^ Xinjiang Medical University, Urumqi, China; ^2^ Department of Oncology Cardiology, Affiliated Cancer Hospital of Xinjiang Medical University, Urumqi, China; ^3^ The Fifth Affiliated Hospital of Xinjiang Medical University, Urumqi, China; ^4^ Department of Oncology Cardiology, Xinjiang Cardiovascular and Cerebrovascular Hospital, Urumqi, China

**Keywords:** epidermal growth factor receptor, EGFR, network meta-analysis, disproportionality analysis, FAERS database, real-world study, pharmacovigilance analysis

## Abstract

**Background:**

Epidermal Growth Factor Receptor Tyrosine Kinase Inhibitors (EGFR-TKIs) in clinical use show promise but can cause AEs, impacting patients’ wellbeing and increasing costs.

**Methods:**

This study utilized two methods: network meta-analysis (NMA) and disproportionality analysis (DA). For NMA, we searched PubMed, Embase, Cochrane Central Register of Controlled Trials, and ClinicalTrials.gov up to 10 September 2024, for phase II/III RCTs comparing EGFR-TKI monotherapy with chemotherapy or other EGFR-TKIs. Using STATA 18.0, we calculated odds ratios (ORs) with 95% confidence intervals (CIs) and assessed heterogeneity via Chi-squared and I^2^ tests. Adverse events (AEs) were ranked using the surface under the cumulative ranking curve (SUCRA). For DA, we analyzed FAERS data (January 2004-June 2024), evaluating AE signals with reporting odds ratios (RORs) and 95% CIs; signals were considered significant if the ROR and its 95% CI lower bound exceeded 1. Primary outcomes for NMA included all-grade AEs, grade ≥3 AEs, specific AEs, and AE-related mortality. For DA, outcomes included EGFR-TKI as the primary AE cause, time from treatment to AE, and AE-related mortality.

**Results:**

NMA: 48% of EGFR-TKI patients experienced AEs, with 32.7% being severe. Afatinib showed highest toxicity; Icotinib was safest. Osimertinib was associated with highest risks of leukopenia (8%) and thrombocytopenia (9%). DA: Osimertinib had strongest links to cardiac diseases and blood/lymphatic disorders. Gefitinib had the strongest signal for interstitial lung diseases; Erlotinib for anorexia. Most AEs occurred within 30 days, but cardiac disorders had a median onset of 41 days. Osimertinib had the highest AE-related mortality, with cardiac disorders leading in fatalities.

**Conclusion:**

This study used NMA and DA to explore EGFR-TKI-related AEs. Drugs varied in AE profiles, mostly mild, but Osimertinib and Dacomitinib were associated with more severe events. Osimertinib carried a high cardiac risk, delayed onset, and high mortality. Thus, comprehensive patient assessment and close monitoring are crucial with EGFR-TKI use.

## 1 Introduction

EGFR is a tyrosine kinase receptor critical for tumor cell proliferation and survival. Upon ligand binding, EGFR becomes activated, forming dimers that stimulate downstream signaling pathways, promoting cell differentiation, proliferation, and potentially carcinogenesis. EGFR overexpression is closely linked to tumor angiogenesis and local metastasis (Sabbah et al., 2020; Sigismund et al., 2018). Approved EGFR Tyrosine Kinase Inhibitors (TKIs), such as Gefitinib, Erlotinib, Lapatinib, and Icotinib, constitute the first-generation EGFR inhibitors. They reversibly bind to the EGFR’s PTK domain, effectively blocking ATP binding and inhibiting EGFR activation and cellular proliferation (Dutta and Maity, 2007; Sabbah et al., 2020; Sigismund et al., 2018). In contrast, second-generation EGFR-TKIs, including Afatinib, Neratinib, and Dacomitinib, covalently bind to EGFR, achieving irreversible kinase inhibition and demonstrating superior efficacy compared to first-generation TKIs (Stasi and Cappuzzo, 2014). The third-generation EGFR inhibitor Osimertinib stands out by forming stable covalent bonds with EGFR harboring the T790M mutation, addressing resistance issues associated with first- and second-generation TKIs (Nagasaka et al., 2021; Li et al., 2022; Dong et al., 2021).

Additionally, Vandetanib, which inhibits kinases beyond EGFR, is classified as a multi-kinase inhibitor. These drugs have been approved for treating various solid tumors, including non-small cell lung cancer (NSCLC), head and neck cancer, pancreatic cancer, and esophageal cancer (Kelly et al., 2015; Dutton et al., 2014; Propper et al., 2014; Harrington et al., 2015). However, they are associated with a range of toxicities, such as diarrhea, rash, mucositis, and fatigue (Zhang et al., 2017; Sheng et al., 2016), significantly impacting patients’ physiological functions and quality of life, leading to reduced adherence and increased treatment costs. Notably, EGFR-TKI toxicity profiles vary across trials, prompting further investigation into this area.

We investigated the characteristics of AEs associated with EGFR-TKIs using NMA and DA based on the FAERS database. NMA, which integrates evidence from multiple studies, provides a comprehensive and indirect assessment of different intervention measures, thereby resolving issues of missing or conflicting evidence and enhancing the reliability of the results (Florez et al., 2024; Zhang et al., 2017; Sheng et al., 2016). DA leverages extensive spontaneous reporting data from the global FAERS database to capture the diversity and complexity of EGFR-TKI-related AEs, promptly identify potential safety issues, and explore the distribution characteristics of these AEs across different populations (Fang et al., 2014; Fusaroli et al., 2024). The real-time updating capability of the FAERS database ensures the timeliness and accuracy of our analysis on EGFR-TKI-related AEs. Through these two approaches, we conducted an in-depth analysis of the characteristics of EGFR-TKI-related AEs.

## 2 Materials and methods

**FIGURE 1:**
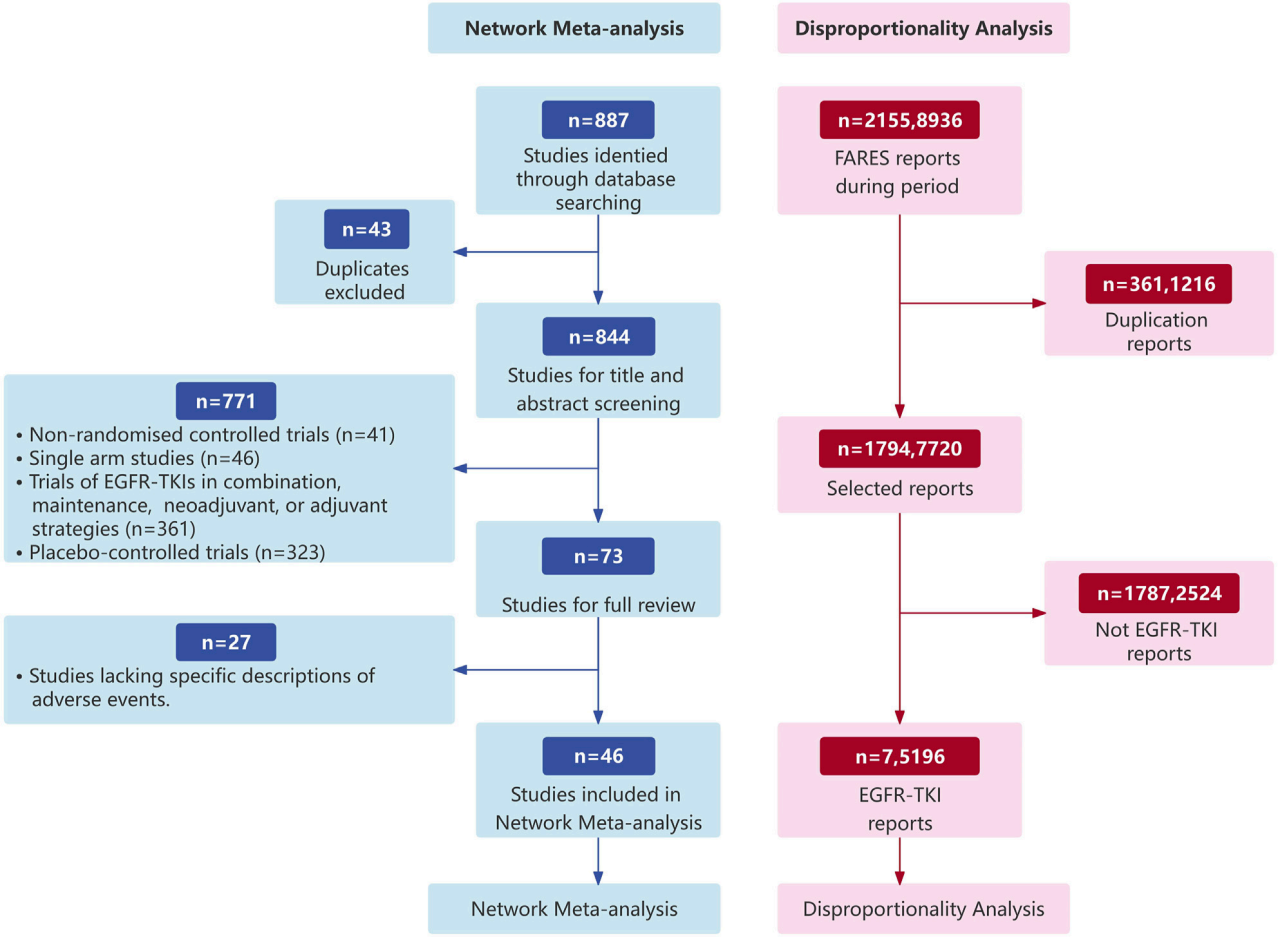
Flow diagram of the study design.

This study employed a hybrid approach, integrating two methodologies: NMA and DA. The latter was grounded in the FAERS database, with the objective of elucidating the characteristics of AEs associated with EGFR-TKI drugs (Figure 1).

### 2.1 Network meta-analysis

#### 2.1.1 Search strategies

We searched the PubMed, Embase, Cochrane Central Register of Controlled Trials, and ClinicalTrials.gov databases using 'NSCLC’ and “EGFR” as primary search terms, limited to RCTs, to identify relevant literature in all languages up to 10 September 2024. Additionally, we examined the reference lists of related articles to find additional studies. The detailed search strategy is presented in Table 1.

**TABLE 1:** PubMed retrieval strategy.

No	Query
#1	“Receptors, Vascular Endothelial Growth Factor” [Mesh]
#2	(((((((((((((((((((Epidermal Growth Factor Receptor Tyrosine Kinase inhibitor [Title/Abstract]) OR (EGFR-TKI [Title/Abstract])) OR (Epidermal Growth Factor Receptor Kinase [Title/Abstract])) OR (Epidermal Growth Factor Receptor Tyrosine Kinase [Title/Abstract])) OR (EGF Receptors [Title/Abstract])) OR (Receptor, Epidermal Growth Factor [Title/Abstract])) OR (VEGF [Title/Abstract])) OR (Vascular Endothelial Growth Factor [Title/Abstract])) OR (VEGF Receptors [Title/Abstract])) OR (Receptors, VEGF [Title/Abstract])) OR (Vascular Endothelial Growth Factor Receptor [Title/Abstract])) OR (VEGF Receptor [Title/Abstract])) OR (Gefitinib [Title/Abstract])) OR (Erlotinib [Title/Abstract])) OR (Icotinib [Title/Abstract])) OR (Afatinib [Title/Abstract])) OR (Dacomitinib [Title/Abstract])) OR (Osimertinib [Title/Abstract])) OR (Vandetanib [Title/Abstract]))
#3	#1 OR #2

#### 2.1.2 Study selection

Inclusion criteria: (1) Phase II or III RCTs comparing the safety of EGFR-TKI monotherapy with chemotherapy or other EGFR-TKIs; (2) Studies must provide detailed data on systemic AEs (including all grades and/or ≥ Grade 3) and/or specific AEs (all grades).

Exclusion criteria: (1) Trials involving EGFR-TKIs in combination therapy, maintenance therapy, neoadjuvant therapy, or adjuvant therapy; (2) Trials comparing EGFR-TKIs with monoclonal antibodies, immunotherapy, certain pathway inhibitors, or other non-conventional chemotherapy methods; (3) Trials involving treatments not approved by any food and drug administration authority; (4) Exclusion of original trial data if safety results have been updated in subsequent data from mature or longer follow-up periods to avoid duplication and obsolescence.

#### 2.1.3 Data extraction

The primary outcomes were all-grade and ≥Grade 3 systemic AEs. Two researchers (T.C. and J.Y.) independently extracted information from each study into a predefined electronic spreadsheet, including baseline characteristics and the number of patients experiencing AEs. AEs designated as treatment-related were preferred; however, if such data were unavailable in the trial, any reported AE data were used instead. Data from supplementary materials were also checked and extracted. When necessary, study authors and pharmaceutical companies were contacted to request complete and updated information.

#### 2.1.4 Risk of bias assessment

The research team (X.Y.L., M.J.G., J.W.L.) independently assessed the risk of bias for each study using the Cochrane Risk of Bias Tool (Higgins et al., 2011; Fang et al., 2014; Fusaroli et al., 2024). The following potential sources of bias were considered: random sequence generation, allocation concealment, blinding of participants and personnel, blinding of outcome assessment, incomplete outcome data, selective reporting, and other biases. Studies were categorized as having a low risk, unclear risk, or high risk of bias. A risk of bias graph was generated using Review Manager version 5.4.

#### 2.1.5 Statistical analysis

All analyses were conducted using STATA 18.0 software. OR and their 95% confidence intervals (95% CI) were calculated to evaluate binary variables. Heterogeneity was assessed using the Chi-squared test and I^2^ statistics. Significant statistical heterogeneity was indicated when I^2^ > 50%, necessitating the use of a fixed-effects model; otherwise, a random-effects model was employed (Peters et al., 2006; Zintzaras and Ioannidis, 2005). Statistical significance was set at P < 0.05. The NMA integrated both direct and indirect evidence. In each loop, IF were used to assess heterogeneity. If the 95% confidence interval of IF included zero, it indicated no significant statistical differences (Song et al., 2003; Peters et al., 2006; Zintzaras and Ioannidis, 2005). Sensitivity analyses were conducted to assess the robustness of the results. The SUCRA was employed to rank AEs associated with EGFR-TKI and chemotherapy, where a higher SUCRA value indicates greater toxicity of the intervention (Salanti et al., 2011; Peters et al., 2006; Zintzaras and Ioannidis, 2005).

### 2.2 Disproportionality analysis

#### 2.2.1 Data collection

The data for this study were obtained from the FAERS database. We downloaded AE reports from the FDA website covering the period from the first quarter of 2004 to the second quarter of 2024. Due to the presence of duplicate reports in the FAERS database, we only utilized the most recent reports for each patient and those that included complete age information. In FAERS, the descriptions of AEs adhere to the Medical Dictionary for Regulatory Activities (MedDRA), established by the International Conference on Harmonization of Technical Requirements for Registration of Pharmaceuticals for Human Use (ICH). For our study, the names of AEs were based on MedDRA version 27.0. EGFR-TKI drugs were defined as the following eight medications: Gefitinib, Erlotinib, Icotinib, Afatinib, Dacomitinib, Osimertinib and Vandetanib, Lapatinib and Neratinib were excluded from this analysis due to its frequent use in combination therapy, which complicates the accurate assessment of AEs for individual drugs. In the FAERS database, AEs are classified at different levels, including “System Organ Classes (SOC)” based on organ systems and “Preferred Terms (PT)” based on specific AEs. We extracted clinical characteristics of the a forementioned drugs, including gender, age, reporting region, and reporter. Additionally, we collected data on the number of AEs, the time elapsed since the initial medication use, and the number and proportion of deaths.

#### 2.2.2 Data deduplication

Due to the self-reporting nature of data collection in the FAERS database, instances of duplicate or withdrawn/deleted reports are common. To address this issue, FDA official guidelines provide specific rules for data deduplication and lists of reports to be deleted. This study rigorously followed the guidelines provided on the FDA’s official website for data cleaning. The deduplication process involved first using the method recommended by the FDA. Specifically, we selected the PRIMARYID, CASEID, and FDA_DT fields from the DEMO table and sorted them by CASEID, FDA_DT, and then PRIMARYID. For records with identical CASEIDs, the one with the most recent FDA_DT was retained; if both CASEID and FDA_DT were the same, the record with the highest PRIMARYID value was kept. Additionally, since the first quarter of 2019, each quarter’s data package includes a list of reports to be deleted. After initial deduplication, these reports were further removed based on their CASEIDs as listed.

#### 2.2.3 Statistical analysis

The DA is used to detect signals of AEs induced by EGFR-TKIs. This analysis compares the proportion of AE reports for EGFR-TKIs with those for all other drugs. The detection of AE signals is evaluated through the ROR and the 95% confidence interval (CI) (Table 2). Specifically, when both the ROR and the lower limit of the corresponding 95% CI are greater than 1, the risk signal is considered significant (Oshima et al., 2018; Hamano et al., 2021). All data analyses were independently conducted by two or more authors. All statistical analyses were performed using SAS 9.4.

**TABLE 2:** Algorithms we used for signal detection.

Algorithms	Equation	Criteria
ROR	$ROR=\frac{\left(a/c\right)}{\left(b/d\right)}=\frac{ad}{bc}$	Lower limit of 95% CI > 1, N ≥ 3
	95%CI = eln (ROR) ± 1.96 (1/a+1/b+1/c+1/d)^0.5^	
Equation		
a: number of reports containing both the target drug and target adverse drug reaction		
b: number of reports containing other adverse drug reaction of the target drug		
c: number of reports containing the target adverse drug reaction of other drugs		
d: number of reports containing other drugs and other adverse drug reactions		
95%CI: 95% confidence interval		
ROR: Reporting Odds Ratio		

## 3 Results

### 3.1 Network meta-analysis

#### 3.1.1 Description of selected studies

Initially, we reviewed a total of 887 potential records from databases. After removing duplicates, 884 records were screened based on their titles and abstracts, and 73 full-text articles were retrieved and reviewed (Figure 1). Ultimately, 46 RCTs met the inclusion criteria, encompassing 15,773 patients who received one of eight different treatments, including Epidermal Growth Factor Receptor Tyrosine Kinase Inhibitors (EGFR TKIs) and chemotherapy. Among these participants, 6,954 (44.4%) were female. The median follow-up duration was 21.0 months. The main characteristics of all studies are reported in Table 3.

**TABLE 3:** Baseline characteristics of studies included in the network meta-analysis.

No	Study (phase)	Tumor type	No. of patients (female%)	Median age (Year)	Treatment	Median follow-up (Months)
1	AURA3, 2020 (III) (Papadimitrakopoulou et al., 2020)	NSCLC	279 (62)	62	Osimertinib 80 mg, QD	23.5
			140 (69)	63	Pemetrexed 500 mg/m2 +Carboplatin AUC = 5/Cisplatin 75 mg/m2, Q3W	20.3
2	LUX-Lung 7, 2017 (IIb) (Paz-Ares et al., 2017)	NSCLC	160 (57)	63	Afatinib 40 mg, QD	42.6
			159 (67)	63	Gefitinib 250 mg, QD	42.6
3	ARCHER1050, 2021 (III) (Cheng et al., 2021)	NSCLC	225 (56)	61	Gefitinib 250 mg, QD	47.9
			227 (64)	62	Dacomitinib 45 mg, QD	47.9
4	WJOG5108L, 2016 (III) (Urata et al., 2016)	NSCLC	276 (54.3)	67	Erlotinib 150 mg, QD	26.5
			275 (54.5)	68	Gefitinib 250 mg, QD	25.1
5	CTONG0901, 2017 (III) (Yang et al., 2017)	NSCLC	128 (53.1)	NG	Erlotinib 150 mg, QD	22.1
			128 (53.9)	NG	Gefitinib 250 mg, QD	22.1
6	LUX-Head and Neck 3,2019 (III) (Guo et al., 2019)	HNSCC	228 (15)	55.5	Afatinib 40 mg, QD	6.4
			112 (12)	58	Methotrexate 40mg/m2, QW	6.4
7	ARCHER1009, 2014 (III) (Ramalingam et al., 2014)	NSCLC	439 (34)	64	Dacomitinib 45 mg, QD	7.1
			439 (37)	62	Erlotinib 150 mg, QD	7.1
8	Kim et al., 2012 (II) (Kim et al., 2012)	NSCLC	48 (85.4)	60	Erlotinib 150 mg, QD	NG
			48 (85.4)	56	Gefitinib 250 mg, QD	NG
9	ISTANA, 2010 (III) (Lee et al., 2010)	NSCLC	82 (32.9)	57	Gefitinib 250 mg, QD	NG
			79 (43)	58	Docetaxel 75mg/m2, Q3W	NG
10	LUX-Head and Neck 1,2015 (III) (Machiels et al., 2015)	HNSCC	322 (15)	60	Afatinib 40 mg, QD	6.7
			161 (15)	59	Methotrexate 40mg/m2, QW	6.7
11	LUX-Lung 8, 2015 (III) (Soria et al., 2015)	LUSC	398 (16)	65	Afatinib 40 mg, QD	6.7
			397 (17)	64	Erlotinib 150 mg, QD	6.7
12	Li et al., 2014 (II) (Li et al., 2014)	LUAD	61 (34.4)	54	Erlotinib 150 mg, QD	14.7
			62 (37.1)	55	Pemetrexed 500 mg/m2, Q3W	14.7
13	TAILOR, 2013 (III) (Garassino et al., 2013)	NSCLC	107 (29.4)	66	Erlotinib 150 mg, QD	33
			104 (33.6)	67	Docetaxel 75 mg/m2, Q3W or 35 mg/m2, Q4W	33
14	IFCT-0301, 2010 (II) (Morère et al., 2010)	NSCLC	43 (11.6)	70	Gefitinib 250 mg, QD	NG
			42 (21.4)	71	Docetaxel 75 mg/m2, Q3W	NG
15	ICOGEN, 2013 (III) (Shi et al., 2013)	NSCLC	200 (41.2)	57	Icotinib 125 mg, TID	NG
			199 (43.4)	57	Gefitinib 250 mg, QD	NG
16	Kim et al., 2016 (II) (Kim et al., 2016)	NSCLC	48 (27.1)	67	Gefitinib 250 mg, QD	60.6
			47 (29.8)	64	Pemetrexed 500 mg/m2, Q3W	60.6
17	Natale et al., 2009 (II) (Natale et al., 2009)	NSCLC	85 (61)	61	Gefitinib 250mg, QD	NG
			83 (58)	63	Vandetanib 300mg, QD	NG
18	PF-00299804, 2012 (II) (Ramalingam et al., 2012)	NSCLC	94 (41)	60	Dacomitinib 45 mg, QD	NG
			94 (40)	62	Erlotinib 150 mg, QD	NG
19	V-15–32, 2008 (III) (Maruyama et al., 2008)	NSCLC	244 (38.4)	NG	Gefitinib 250 mg, QD	21
			239 (38.1)	NG	Docetaxel 60 mg/m2, Q3W	21
20	Stewart et al., 2009 (II) (Stewart et al., 2009)	HNSCC	158 (16)	NG	Gefitinib 250mg, QD	NG
			161 (16)	NG	Methotrexate 40mg/m2, QW	NG
21	SIGN, 2006 (II) (Cufer et al., 2006)	NSCLC	68 (30.8)	63	Gefitinib 250 mg, QD	9.2
			71 (30.1)	60	Docetaxel 75 mg/m2, Q3W	9.4
22	ENSURE, 2015 (III) (Wu et al., 2015)	NSCLC	110 (61.8)	58	Erlotinib 150 mg, QD	28.9
			104 (60.7)	56	Gemcitabine 1250 mg/m2 +Cisplatin 75 mg/m2, Q3W	27.1
23	HORG, 2013 (III) (Karampeazis et al., 2013)	NSCLC	166 (18.7)	65	Erlotinib 150 mg, QD	29
			166 (16.9)	66	Pemetrexed 500 mg/m2, Q3W	27.3
24	Lilenbaum et al., 2008 (II) (Lilenbaum et al., 2008)	NSCLC	52 (55.8)	NG	Erlotinib 150 mg, QD	NG
			51 (45.1)	NG	Carboplatin AUC = 6 +Paclitaxel 200 mg/m2, Q3W	NG
25	OPTIMAL, 2011 (III) (Zhou et al., 2011)	NSCLC	83 (58.5)	57	Erlotinib 150 mg, QD	15.6
			72 (59.7)	59	Gemcitabine 1000 mg/m2 +Cisplatin AUC = 5, Q3W	15.6
26	IPASS, 2009 (III) (Mok et al., 2009)	NSCLC	607 (79.5)	57	Gefitinib 250 mg, QD	5.6
			589 (79.1)	57	Carboplatin AUC = 5/6 +Paclitaxel 200 mg/m2, Q3W	5.6
27	KCSG-LU08-01, 2012 (III) (Sun et al., 2012)	NSCLC	68 (85.3)	58	Gefitinib 250 mg, QD	15.9
			67 (85.1)	64	Pemetrexed 500 mg/m2, Q3W	15.9
28	INTEREST, 2008 (III) (Kim et al., 2008)	NSCLC	729 (36.4)	61	Gefitinib 250 mg, QD	7.6
			715 (33.4)	60	Docetaxel 75 mg/m2, Q3W	7.6
29	DELTA, 2014 (III) (Kawaguchi et al., 2014)	NSCLC	150 (28.0)	68	Erlotinib 150 mg, QD	8.9
			150 (29.1)	67	Docetaxel 60 mg/m2, Q3W	8.9
30	TITAN, 2012 (III) (Ciuleanu et al., 2012)	NSCLC	196 (20.7)	59	Erlotinib 150 mg, QD	27.9
			213 (27.6)	59	standard docetaxel or pemetrexed dosing schedule	24.8
31	WJTOG3405, 2010 (III) (Mitsudomi et al., 2010)	NSCLC	87 (68.6)	64	Gefitinib 250 mg, QD	2.7
			88 (69.8)	64	Cisplatin 80 mg/m2 +Docetaxel 60 mg/m2, Q3W	2.7
32	EURTAC, 2012 (III) (Rosell et al., 2012)	NSCLC	84 (67.4)	65	Erlotinib 150 mg, QD	18.9
			82 (78.2)	65	Docetaxel 75 mg/m2 or gemcitabine 1250 mg/m2 +Cisplatin 75 mg/m2, Q3W	14.4
33	Heigener, 2014 (II) (Heigener et al., 2014)	NSCLC	144 (32.4)	76	Erlotinib 150 mg, QD	NG
			140 (32.4)	76	Carboplatin AUC = 5+Vinorelbine 25 mg/m2 on days 1 and 8, Q3W	NG
34	CONVINCE, 2017(III) Shi et al., 2017	NSCLC	148 (70.9)	56	Icotinib 125 mg, TID	NG
			137 (69.3)	56	Cisplatin 75 mg/m2 +Pemetrexed 500 mg/m2, Q3W	NG
35	Han et al., 2017 (II) (Han et al., 2017)	NSCLC	41 (56.1)	NG	Gefitinib 250 mg, QD	NG
			40 (57.5)	NG	Pemetrexed 500 mg/m2 + Carboplatin AUC = 5, Q4W, 6 cycles	NG
36	IFCT-0504, 2015 (II) (Cadranel et al., 2015)	NSCLC	66 (38.8)	67	Erlotinib 150 mg, QD	69.4
			66 (39.4)	68	Paclitaxel 90 mg/m2 + Carboplatin AUC = 6, Q4W	69.4
37	LUX-Lung 6, 2014(III) (Wu et al., 2014)	NSCLC	242 (64)	58	Afatinib 40 mg, QD	16.6
			122 (68)	58	gemcitabine 1000 mg/m^2^, on day 1 and day 8+ cisplatin 75 mg/m^2^, on day 1	16.6
38	CTONG0806, 2014 (II) (Zhou et al., 2014)	NSCLC	81 (33.3)	58	Gefitinib 250 mg, QD	10.6
			76 (38.2)	56	Pemetrexed 500 mg/m2, Q3W	10.6
39	PROSE, 2014 (III) (Gregorc et al., 2014)	NSCLC	134 (26.1)	66	Erlotinib 150 mg, QD	32.4
			129 (29.5)	64	Pemetrexed 500 mg/m2 or Docetaxel 75mg/m2, Q3W	32.4
40	LUX-Lung3, 2013 (III) (Sequist et al., 2013)	NSCLC	229 (63.9)	62	Afatinib 40 mg, QD	16.4
			111 (67.0)	61	Pemetrexed 500 mg/m2 + Cisplatin 75 mg/m2, Q3W	16.4
41	First-SIGNAL, 2012 (III) (Han et al., 2012)	LUAD	159 (88.0)	57	Gefitinib 250 mg, QD	35
			150 (89.3)	57	Gemcitabine 1250 mg/m2 + Cisplatin 80 mg/m2, Q3W	35
42	Kelly et al., 2012 (IIb) (Kelly et al., 2012)	NSCLC	101 (32.6)	62	Erlotinib 150 mg, QD	NG
			97 (31.0)	63	Pralatrexate 190 mg/m2, Q4W	NG
43	Maemondo et al., 2010 (III) (Maemondo et al., 2010)	NSCLC	114 (63.2)	63.9	Gefitinib 250 mg, QD	17.6
			114 (64)	62.6	Paclitaxel 200 mg/m2 + Carboplatin AUC = 6, Q3W	17.6
44	INVITE, 2008 (II) (Crinò et al., 2008)	NSCLC	94 (22.7)	74	Gefitinib 250 mg, QD	6.4
			96 (26.3)	74	Vinorelbine 30 mg/m2, Q3W	6.2
45	ZEST,2011(III) (Natale et al., 2011)	NSCLC	623 (39)	61	Vandetanib 300mg, QD	7
			617 (36)	61	Erlotinib 150 mg, QD	7
46	BATTLE,2011(II) (Kim et al., 2011)	NSCLC	59(NG)	NG	Erlotinib 150 mg, QD	NG
			54(NG)	NG	Vandetanib 300mg, QD	NG

NSCLC, non-small cell lung cancer.

HNSCC, head and neck squamous cell carcinomas.

LUSC, lung squamous cell carcinoma.

LUAD, lung adenocarcinoma.

NG, not given.

**FIGURE 2:**
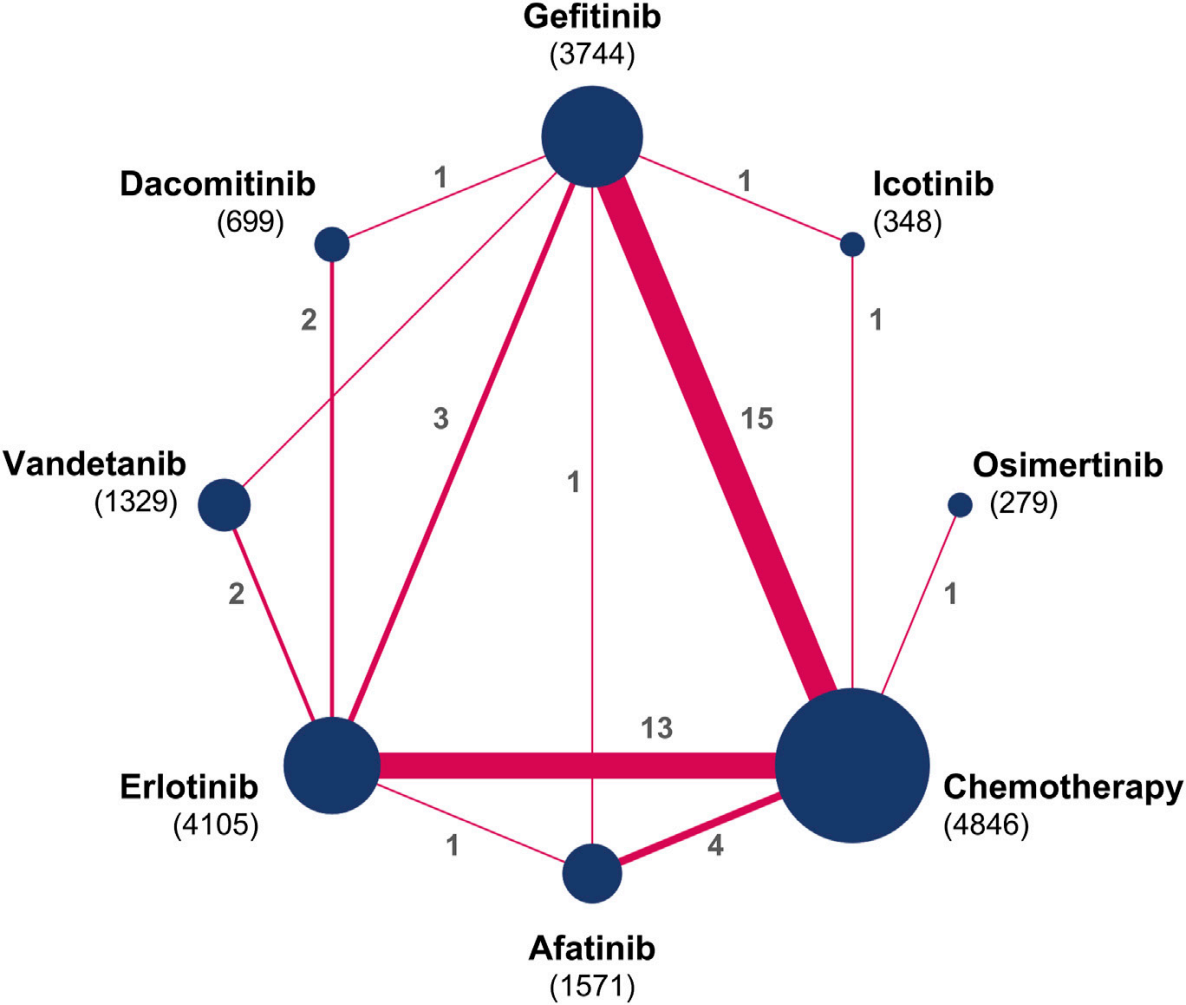
Network diagrams for comparisons on systemic AEs. Circular nodes denote treatments, with each node’s size proportional to the total number of patients (in parentheses) assigned to that treatment. Lines signify direct comparisons; the width of each line corresponds to the number of trials (indicated beside the line) examining the respective comparison.

**FIGURE 3:**
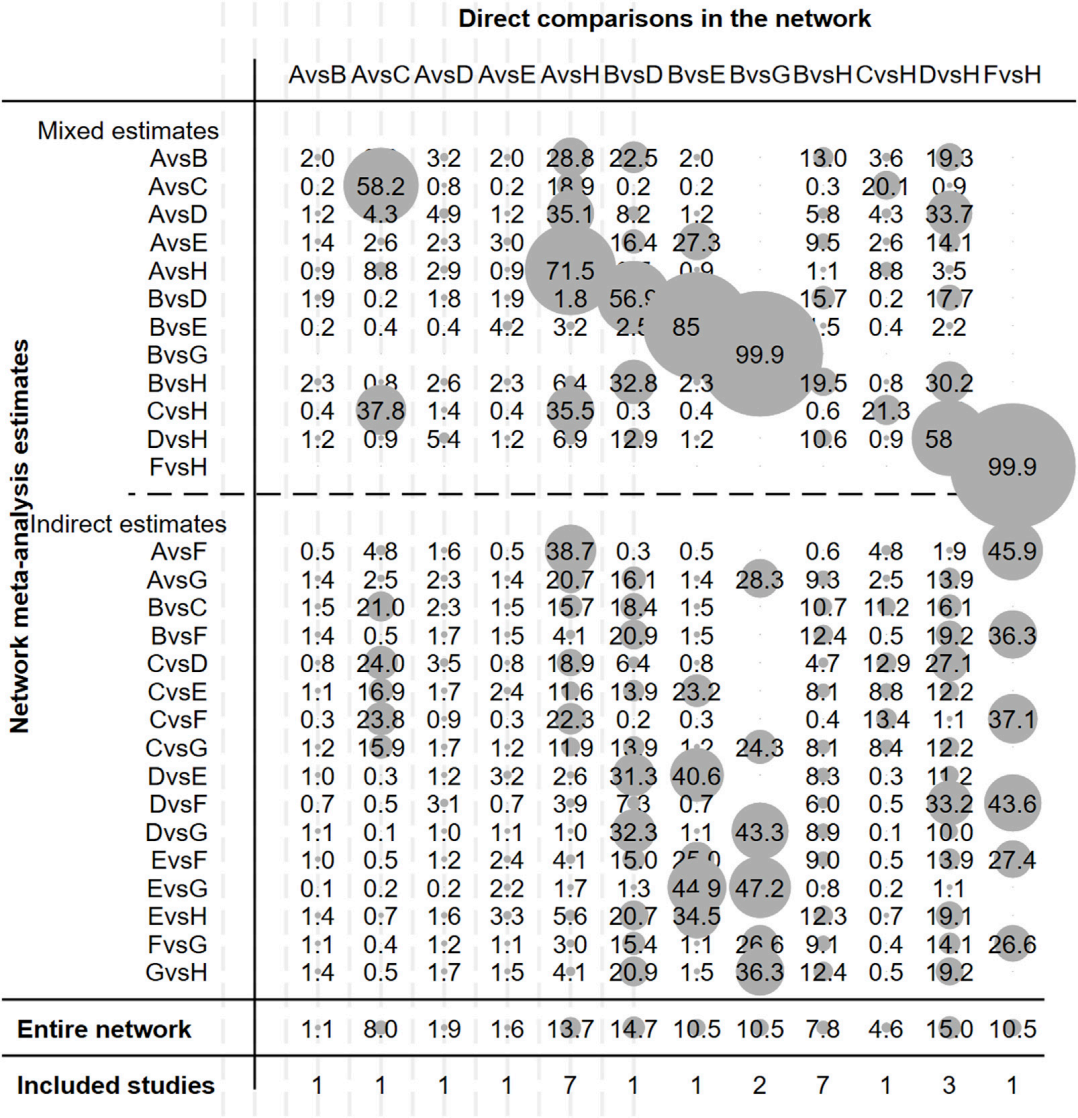
Contribution plot of studies included in this network meta-analysis. Note: A, Gefitinib; B, Erlotinib; C, Icotinib; D, Afatinib; E, Dacomitinib; F, Osimertinib; G, Vandetanib; H, Chemotherapy.

**FIGURE 4:**
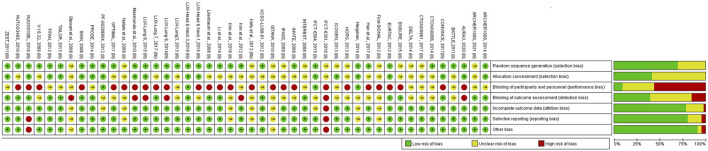
Summary of results from assessment of studies using the Cochrane risk of bias tool.

Figure 2 illustrates the evidence network. Figure 3 depicts the impact of each direct comparison on the overall effect estimate within the network. Figure 4 provides a comprehensive assessment of the risk of bias, with the primary sources of high risk being related to participant and personnel blinding, largely due to the considerable proportion of open-label studies.

#### 3.1.2 Overview

Among the studies reviewed, 26 reported on the number of patients experiencing any grade of systemic AEs (AEs), while 32 documented those experiencing at least one grade 3 or higher AE. In the chemotherapy group, 2,221 patients (82.1%) experienced all-grade AEs, and 1,794 patients (46.1%) had grade 3 or higher AEs. Over 100 different types of specific AEs were reported based on their incidence and clinical relevance. Among them, 20 AEs of interest were identified, including Rash, Alopecia, Fatigue, Dry skin, Stomatitis, Anorexia, Nausea/Vomiting, Constipation, Myalgia/Arthralgia, Aspartate aminotransferase (AST) increased, Alanine aminotransferase (ALT) increased, Creatinine increased, Anemia, White blood cell decreased, Platelet count decreased, Dyspnea, Pneumonia, Insomnia, Chest pain, and Interstitial lung disease (ILD).

#### 3.1.3 Systemic AEs

In a cohort of 5671 patients undergoing EGFR-TKI therapy, 48% reported experiencing at least one systemic AEs. Of these, 32.7% encountered AEs of grade three or higher severity. In contrast, within the chemotherapy group, the total incidence of any-grade AEs was 2221 individuals (82.1%), with 1794 (46.1%) suffering from AEs of grade three or above, as summarized in the Table 4. Notably, The study involving Vandetanib did not report data on the number of participants experiencing at least one systemic AE.

**TABLE 4:** Number and incidence of AEs induced by different drugs.

	All grade AE			≥3grade AE		
	AEs Count	Total participants	Adverse event rate	AEs Count	Total participants	Adverse event rate
Gefitinib	1,797	2,233	80.5%	727	3,253	22.3%
Erlotinib	1,502	1,767	85.0%	459	5,655	8.1%
Afatinib	1,263	1,342	94.1%	515	1,571	32.8%
Icotinib	201	384	57.8%	21	384	5.5%
Dacomitinib	579	606	95.5%	252	606	41.6%
Osimertinib	275	279	98.6%	104	279	37.3%
Vandetanib	NA	NA	NA	NA	NA	NA
EGFR-TIK	5,617	6,611	85.0%	2,078	11,748	17.7%
Chemotherapy	2,221	2,705	82.1%	1,794	3,890	46.1%

In terms of systemic all-grade AEs (Table 5, lower triangle), Afatinib induced the most frequent toxicity. Compared to Osimertinib, Afatinib showed significant differences in systemic all-grade AEs with other drugs, including chemotherapy. Dacomitinib was the second most toxic, significantly differing from Gefitinib and Icotinib. Among these, Icotinib was the safest EGFR-TKI, showing significant differences with all drugs except Osimertinib; Gefitinib was the second safest, significantly different from Erlotinib and Osimertinib.

**TABLE 5:** Pooled estimates of the network meta-analysis.

All-grade AEs	≥Grade 3 AEs					
Gefitinib	1.47 (0.90, 2.38)	0.67 (0.25, 1.78)	**2.79 (1.58, 4.95)**	2.27 (0.95, 5.45)	2.63 (0.76, 9.17)	**4.06 (2.87, 5.73)**
0.61 (0.33, 1.12)	**Erlotinib**	0.46 (0.16, 1.31)	**1.90 (1.05, 3.46)**	1.55 (0.65, 3.70)	1.80 (0.51, 6.38)	**2.77 (1.83, 4.19)**
**2.53 (1.24, 5.15)**	**4.15 (1.76, 9.78)**	**Icotinib**	**4.18 (1.40, 12.53)**	3.41 (0.93, 12.48)	3.95 (0.84, 18.63)	**6.08 (2.27,16.30)**
**0.20 (0.11, 0.38)**	**0.34 (0.18, 0.61)**	**0.08 (0.03, 0.19)**	**Afatinib**	0.81 (0.30, 2.20)	0.94 (0.26, 3.46)	1.45 (0.88, 2.41)
**0.33 (0.12, 0.92)**	0.54 (0.22, 1.32)	**0.13 (0.04,0.43)**	**1.61 (0.56, 4.61)**	**Dacomitinib**	1.16 (0.26, 5.15)	1.78 (0.73, 4.34)
1.16 (0.11, 12.53)	1.91 (0.17, 21.02)	0.46 (0.04,5.36)	5.69 (0.51, 62.99)	3.53 (0.28, 44.91)	**Osimertinib**	1.54 (0.46, 5.11)
**0.59 (0.41, 0.86)**	0.97 (0.59, 1.60)	**0.23 (0.11, 0.48)**	**2.90 (1.71, 4.90)**	1.80 (0.67, 4.80)	0.51 (0.05, 5.32)	**Chemotherapy**

The numbers in the cells are odds ratios, with 95% confidence intervals in parentheses. If the number is greater (less) than 1, it indicates that the treatment defined by the column is more (less) toxic. The bold numbers demonstrate a statistically significant difference in adverse event toxicity between the two drugs.

In the context of AEs of grade≥3 (Table 5, upper triangle), chemotherapy exhibits the highest toxicity, significantly differing from Gefitinib, Erlotinib, and Icotinib. Among EGFR-TKIs, Afatinib is identified as the most toxic, also markedly different from Gefitinib, Erlotinib, and Icotinib, followed by Osimertinib. Notably, Icotinib stands out as the safest EGFR-TKI, with Gefitinib ranking second in terms of safety.

Comparative analysis demonstrated distinct safety profiles across EGFR-TKI generations. Among first-generation agents, icotinib exhibited a significantly lower risk of all-grade adverse events (AEs) compared to gefitinib and erlotinib (p < 0.05), whereas no statistically significant difference was observed between gefitinib and erlotinib. Although numerical variations existed in grade ≥3 AEs among the three agents, none achieved statistical significance. Within second-generation EGFR-TKIs, afatinib demonstrated a higher incidence of grade ≥3 AEs relative to dacomitinib (p = 0.02), while all-grade AE rates showed no inter-agent statistical disparity.

**FIGURE 5:**
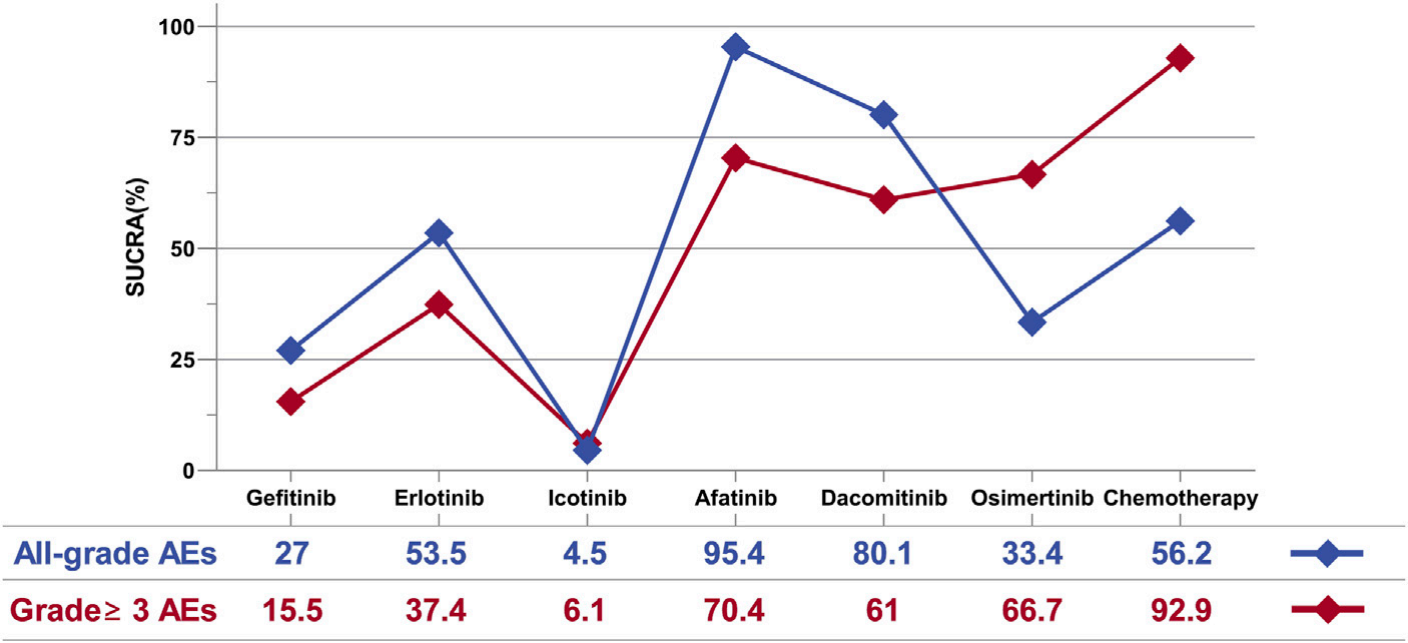
Surface under the cumulative ranking curve for AEs.

We ranked the drugs based on their Surface Under the Cumulative Ranking (SUCRA) values, as illustrated in Figure 5. For all-grade AEs, the ranking from highest to lowest toxicity is as follows: Afatinib (SUCRA = 95.4%), Dacomitinib (80.1%), Chemotherapy (56.2%), Erlotinib (53.5%), Osimertinib (33.4%), Gefitinib (27%), and Icotinib (4.5%). For grade 3 and higher AEs, the ranking is: Chemotherapy (92.9%), Afatinib (70.4%), Osimertinib (66.7%), Dacomitinib (61%), Erlotinib (37.4%), Gefitinib (15.5%), and Icotinib (6.1%).

#### 3.1.4 Specific AEs

**FIGURE 6:**
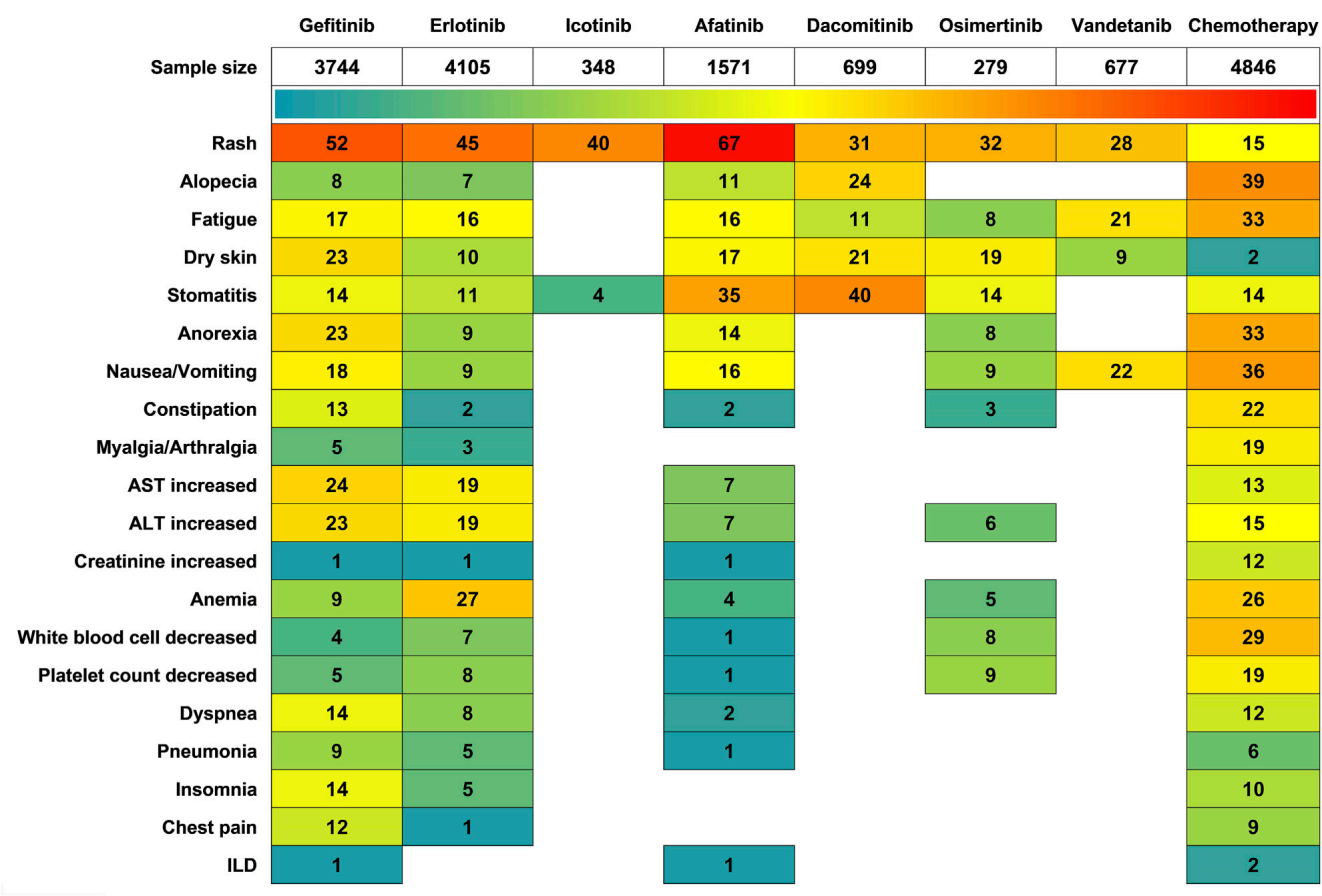
Heatmap of AEs Incidence Induced by different treatments.

We conducted a statistical analysis of the incidence rates of AEs across all grades (Figure 6). Among all EGFR-TKI-induced AEs, rash had the highest incidence rate. Afatinib led to a 67% incidence rate of rash, followed by Gefitinib at 52%, and Vandetanib had the lowest incidence rate at 28%. Regarding Hepatic insufficiency, Gefitinib exhibited higher incidence rates than other EGFR-TKIs and chemotherapy, with an AST increased incidence rate of 24% and alanine ALT increased at 23%. Erlotinib had the next highest rates, with both AST and ALT increased at 19%. In terms of hematologic AEs, Erlotinib had a significantly higher anemia incidence rate at 27%, slightly above the chemotherapy group’s 26%. For leukopenia and thrombocytopenia, Osimertinib showed the highest incidence rates among EGFR-TKIs at 8% and 9%, respectively, but these were still much lower than the chemotherapy group’s 29% and 19%. Additionally, chest pain was only reported in patients treated with Gefitinib and Erlotinib, with incidence rates of 12% and 1%, respectively, compared to 9% in the chemotherapy group. Notably, both Gefitinib and Afatinib had an interstitial lung disease (ILD) incidence rate of 1%, while no reports were made for the other EGFR-TKIs.

Comprehensive safety evaluations revealed distinct toxicity patterns across EGFR-TKI generations. Regarding first-generation agents, icotinib demonstrated superior tolerability with significantly lower incidence rates of most adverse events (AEs) compared to both gefitinib and erlotinib. Notably, erlotinib exhibited higher frequencies of hematological toxicities (e.g., anemia, leukopenia, thrombocytopenia) than gefitinib, while maintaining comparable or marginally lower rates in other non-hematological AEs. In the second-generation class, dacomitinib showed advantageous AE profiles over afatinib for common toxic effects including rash, fatigue, and nausea/vomiting, though it demonstrated elevated risks of alopecia, xerosis cutis, and stomatitis. These inter-agent contrasts emphasize the necessity for personalized selection of second-generation TKIs based on patients’ susceptibility to specific toxicities and individual quality-of-life priorities.

We compared the specific AEs of interest across different EGFR-TKIs through OR (Table 6), Afatinib has a significantly higher risk of oral mucositis compared to Gefitinib (OR = 5.49), Erlotinib (OR = 4.97), and all other EGFR-TKIs, including Icotinib (OR = 8.01), Dacomitinib (OR = 1.61), and Osimertinib (OR = 4.17). Afatinib also exhibits higher risks of increased creatinine levels, pneumonia, and rash than those reported for other EGFR-TKIs. Osimertinib poses a significant risk of leukopenia, which is higher than that of Vandetanib (OR = 10.25) and Afatinib (OR = 11.41), as well as Gefitinib (OR = 6.23) and Icotinib (OR = 10.66). Similarly, Osimertinib’s risk of thrombocytopenia is greater than that of Gefitinib (OR = 1.74), Erlotinib (OR = 3.13), and Afatinib (OR = 8.8). Gefitinib shows a higher risk of nausea/vomiting compared to other EGFR-TKIs: Erlotinib (OR = 1.85), Icotinib (OR = 1.72), Afatinib (OR = 1.52), Dacomitinib (OR = 1.44), Osimertinib (OR = 3.23), and Vandetanib (OR = 2.08). Additionally, Gefitinib has a higher risk of AST increased and ILD compared to Erlotinib and Afatinib, with no reports of this AE for other EGFR-TKIs. Erlotinib has a higher risk of ALT increased compared to Gefitinib (OR = 1.09), Afatinib (OR = 4.35), Dacomitinib (OR = 2.94), and Osimertinib (OR = 1.96). Furthermore, Erlotinib presents a higher risk of dyspnea compared to Gefitinib (OR = 2.03), Afatinib (OR = 2.33), and Vandetanib (OR = 1.08). Dacomitinib is associated with a higher risk of dry skin among the studied EGFR-TKIs. Vandetanib carries a higher risk of anemia compared to Gefitinib (OR = 2.56), Erlotinib (OR = 2.81), Afatinib (OR = 4.09), and Osimertinib (OR = 5.55). Conversely, Icotinib appears to be relatively safer regarding the a forementioned AEs.

**TABLE 6:** Toxicity estimates regarding specific all-grade AEs in network meta-analysis.

	Rash	Alopecia	Fatigue	Dry skin	Stomatitis	Anorexia	Nausea/Vomiting	Constipation	Myalgia/Arthralgia	AST increased
Vs. Gefitinib										
Erlotinib	1.18	3.77	1.63	1.03	1.1	1.03	0.54	0.71	3.19	0.71
Icotinib	0.69				0.69	0.85	0.58			
Afatinib	3.82	0.67	1.25	0.88	**5.49**	1.03	0.66	0.29		0.31
Dacomitinib	1.25	2.15	1.21	1.26	3.4	1.27	0.69			0.41
Osimertinib	1.16		0.81	1.24	1.33	0.48	0.31	0.32		
Vandetanib	0.58		1.68	0.63		0.97	0.48	0.43		
Chemotherapy	0.14	**15.07**	**3**	**0.08**	1.29	**2.46**	**2.95**	**2.37**	**6.84**	0.73
Vs. Erlotinib										
Icotinib	0.58				0.62	0.82	1.09			
Afatinib	3.23	0.18	0.77	0.85	**4.97**	1	1.22	0.4		0.44
Dacomitinib	1.06	0.57	0.75	1.23	3.08	1.23	1.28			0.58
Osimertinib	0.98		0.5	1.21	1.2	0.46	0.58	0.44		
Vandetanib	0.49		1.03	**0.61**		0.95	0.9	0.61		
Chemotherapy	0.12	**4**	**1.85**	**0.08**	1.17	**2.39**	**5.49**	**3.32**	2.14	1.03
Vs. Icotinib										
Afatinib	5.55				8.01	1.21	1.12			
Dacomitinib	1.82				4.97	1.5	1.18			
Osimertinib	1.69				1.94	0.56	0.53			
Vandetanib	0.84					1.15	0.83			
Chemotherapy	0.2				1.88	2.9	5.05			
Vs. Afatinib										
Dacomitinib	0.33	3.22	0.97	1.44	0.62	1.24	1.05			1.33
Osimertinib	0.3		0.65	1.42	0.24	0.47	0.48	1.09		
Vandetanib	0.15		1.34	0.72		0.95	0.74	1.5		
Chemotherapy	0.04	**22.54**	**2.39**	**0.09**	**0.23**	**2.39**	**4.49**	**8.21**		2.35
Vs. Dacomitinib										
Osimertinib	0.93		0.67	0.98	**0.39**	0.38	0.45			
Vandetanib	0.46		1.38	**0.5**		0.77	0.7			
Chemotherapy	0.11	7	2.47	**0.06**	0.38	1.93	4.28			1.77
Vs. Osimertinib										
Vandetanib	0.5		2.07	0.51		2.04	1.55	1.37		
Chemotherapy	0.12		3.71	**0.06**	0.97	**5.15**	9.44	**7.5**		
Vs. Vandetanib										
Chemotherapy	0.24		1.79	**0.12**		2.53	6.11	5.46		

	ALT increased	Creatinine increased	Anemia	White blood cell decreased	Platelet count decreased	Dyspnea	Pneumonia	Insomnia	Chest pain	ILD
Vs. Gefitinib										
Erlotinib	1.09	1.65	0.91	0.61	0.56	2.03	1.14	0.76	8.57	0.91
Icotinib				0.58						
Afatinib	0.25	12.47	0.63	0.55	0.2	0.88	4.03			0.32
Dacomitinib	0.37									
Osimertinib	0.56		0.46	6.23	1.74					
Vandetanib			2.56			1.89				
Chemotherapy	0.97	12.25	**3.03**	**10.4**	**3.59**	1.06	0.92	0.93	**1.83**	0.77
Vs. Erlotinib										
Icotinib				0.96						
Afatinib	0.23	7.57	0.69	0.9	0.36	0.43	3.55			0.35
Dacomitinib	0.34									
Osimertinib	0.51		0.51	**10.25**	3.13					
Vandetanib			2.81			0.93				
Chemotherapy	0.89	7.44	**3.33**	**17.1**	**6.47**	**0.52**	0.81	1.22	0.21	0.84
Vs. Icotinib										
Afatinib				0.93						
Dacomitinib										
Osimertinib				10.66						
Vandetanib										
Chemotherapy				**17.79**						
Vs. Afatinib										
Dacomitinib	1.49									
Osimertinib	2.23		0.74	**11.41**	8.8					
Vandetanib			4.09			2.15				
Chemotherapy	3.87	0.98	**4.84**	**19.05**	**18.21**	1.2	0.23			2.38
Vs. Dacomitinib										
Osimertinib	1.49									
Vandetanib										
Chemotherapy	2.59									
Vs. Osimertinib										
Vandetanib			5.55							
Chemotherapy	1.74		6.56	1.67	2.07					
Vs. Vandetanib										
Chemotherapy			1.18			0.56				

Numbers in cells are odds ratios and those in bold represent statistically significant results.

### 3.2 Disproportionality analysis

#### 3.2.1 Descriptive analysis

Between January 2004 and June 2024, the FAERS database documented a total of 75,196 patients experiencing AEs from EGFR-TKIs, amounting to 204,092 individual event occurrences. The search process is illustrated in Figure 1. Of these cases, Gefitinib was implicated in 7,184 instances, Erlotinib in 40,159, Afatinib in 5,842, Dacomitinib in 564, Osimertinib in 20,103, and Vandetanib in 1,344. Icotinib, not identified as a direct suspect in any known events, was excluded from this study. The demographic distribution comprised 38,958 females (51.81%), 29,286 males (38.95%), with the majority aged over 65 years (23,749 individuals, 31.58%), followed by those aged between 18 and 64 years (15,328 individuals, 20.38%). The median age was 68 years. Reports predominantly originated from the United States (50.71%), followed by Japan (7.68%). Over one-third of the submissions were made by physicians (Table 7).

**TABLE 7:** Characteristics of patients with AEs associated with EGFR-TKI in FAERS database.

	Gefitinib	Erlotinib	Afatinib	Dacomitinib	Osimertinib	Vandetanib	EGFR-TKI
	n = 7184	n = 40159	n = 5842	n = 564	n = 20103	n = 1344	n = 75196
Gender							
Female (%)	3919 (54.55)	20114 (50.09)	3089 (52.88)	248 (43.97)	11056 (55.00)	532 (39.58)	38958 (51.81)
Male (%)	2706 (37.67)	17745 (44.19)	1951 (33.40)	230 (40.78)	5979 (29.74)	675 (50.22)	29286 (38.95)
Missing (%)	559 (7.78)	2300 (5.73)	802 (13.73)	86 (15.25)	3068 (15.26)	137 (10.19)	6952 (9.25)
Age							
<18 (%)	20 (0.28)	55 (0.14)	14 (0.24)	0	7 (0.03)	18 (1.34)	114 (0.15)
18–64 (%)	2182 (30.37)	6935 (17.27)	1632 (27.94)	223 (39.54)	3738 (18.59)	618 (45.98)	15328 (20.38)
≥65 (%)	3074 (42.79)	10601 (26.40)	2281 (39.04)	220 (39.01)	7137 (35.5)	436 (32.44)	23749 (31.58)
Missing (%)	1908 (26.56)	22568 (56.20)	1915 (32.78)	121 (21.45)	9221 (45.87)	272 (20.24)	36005 (47.88)
Median (Q1,Q3)	67 (59.75)	68 (59.76)	67 (58.74)	64 (56.72)	70 (61.77)	61 (49.70)	68 (59.76)
Country							
United States (%)	294 (4.09)	26401 (65.74)	2016 (34.51)	66 (11.70)	4937 (24.56)	663 (49.33)	38134 (50.71)
Japan (%)	585 (8.14)	911 (2.27)	1120 (19.17)	26 (4.61)	1658 (8.25)	47 (3.50)	5775 (7.68)
China (%)	1103 (15.35)	1373 (3.42)	263 (4.50)	120 (21.28)	576 (2.87)	6 (0.45)	4324 (5.75)
France (%)	191 (2.66)	387 (0.96)	173 (2.96)	0	323 (1.61)	48 (3.57)	1499 (1.99)
Germany (%)	56 (0.78)	362 (0.90)	401 (6.86)	2 (0.35)	141 (0.70)	19 (1.41)	1134 (1.51)
Other (%)	4955 (68.97)	10725 (26.71)	1869 (31.99)	350 (62.06)	12468 (62.02)	561 (41.74)	24330 (32.36)
Reporter type							
Physician (%)	2662 (37.05)	10585 (26.36)	3471 (59.41)	145 (25.71)	5465 (27.18)	487 (36.24)	22815 (30.34)
Pharmacist (%)	642 (8.94)	2407 (5.99)	614 (10.51)	83 (14.72)	2619 (13.03)	99 (7.37)	6464 (8.60)
Other health-professional (%)	793 (11.04)	4360 (10.86)	409 (7.00)	7 (1.24)	446 (2.22)	104 (7.74)	6119 (8.14)
Consume (%)	1054 (14.67)	22200 (55.28)	1313 (22.48)	319 (56.56)	8108 (40.33)	543 (40.40)	33537 (44.60)
Missing (%)	2033 (28.30)	607 (1.51)	35 (0.60)	10 (1.77)	3465 (17.24)	111 (8.26)	6261 (8.33)

#### 3.2.2 Systemic AEs

**FIGURE 7:**
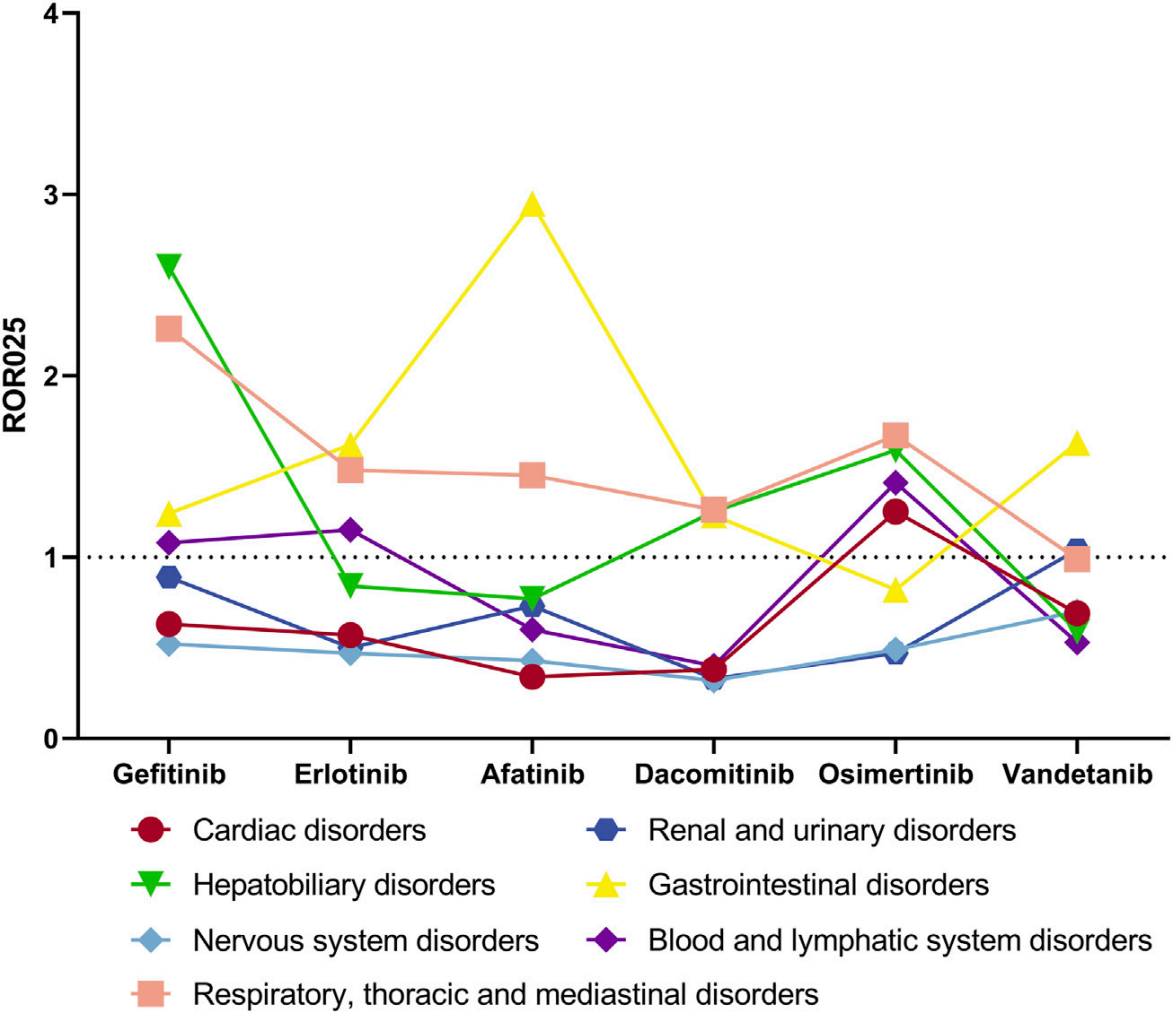
Differences in ROR025 for AEs of interest among EGFR-TKIs at SOC level.

In order to analyze systemic AEs at the SOC level, we quantified the incidence of AEs and computed the ROR as an indicator of signal strength. Based on these metrics and their clinical relevance, we identified specific SOCs for further investigation as prioritized systemic AEs (Table 8). These include disorders of the Blood and Lymphatic System, Cardiac Disorders, Gastrointestinal Disorders, Renal and Urinary Disorders, Respiratory, Thoracic and Mediastinal Disorders, Nervous System Disorders, and Hepatobiliary Disorders. Osimertinib was associated with the strongest signals in Cardiac Disorders (n = 1429, ROR025 = 1.25) and Blood and Lymphatic System Disorders (n = 1034, ROR025 = 1.41); additional positive signals were noted for Respiratory and Hepatobiliary Disorders. Gefitinib exhibited the strongest signals in Respiratory, Thoracic and Mediastinal Disorders (n = 2358, ROR025 = 2.26) and Hepatobiliary Disorders (n = 576, ROR025 = 2.6), with positive signals also observed for Blood and Lymphatic System Disorders and Gastrointestinal Disorders. Afatinib showed the strongest signal in Gastrointestinal Disorders (n = 4798, ROR025 = 2.95), with a positive signal in Respiratory, Thoracic and Mediastinal Disorders. Vandetanib had the strongest signal in Renal and Urinary Disorders (n = 121, ROR025 = 1.04), with a positive signal in Gastrointestinal Disorders. Erlotinib was linked to the highest number of cases in Nervous System Disorders (n = 4794, ROR025 = 0.47), with positive signals in Blood and Lymphatic System Disorders, Gastrointestinal Disorders, and Respiratory Disorders. Dacomitinib demonstrated positive signals in Gastrointestinal, Respiratory, and Hepatobiliary Disorders. Figure 7 illustrates the differences in ROR025 levels across different drugs and SOCs.

**TABLE 8:** Signal profiles of AEs induced by EGFR-TKIs at the SOC level.

SOC	Gefitinib (N = 22653)			Erlotinib (N = 111361)			Afatinib (N = 21758)		
	N	ROR (95%CI)		N	ROR (95%CI)		N	ROR (95%CI)	
Blood and lymphatic system disorders	452	1.19 (1.08–1.31)	◉	2238	1.2 (1.15–1.25)	◉	250	0.68 (0.6–0.77)	◉
Cardiac disorders	419	0.69 (0.63–0.76)	◉	1783	0.6 (0.57–0.62)	◉	227	0.39 (0.34–0.44)	◉
Gastrointestinal disorders	2432	1.29 (1.24–1.35)	◉	14762	1.64 (1.62–1.67)	◉	4798	3.04 (2.95–3.14)	◉
Renal and urinary disorders	424	0.98 (0.89–1.07)	◉	1147	0.53 (0.5–0.56)	◉	342	0.82 (0.73–0.91)	◉
Respiratory, thoracic and mediastinal disorders	2358	2.35 (2.26–2.46)	◉	7737	1.51 (1.48–1.55)	◉	1525	1.53 (1.45–1.61)	◉
Nervous system disorders	1119	0.56 (0.52–0.59)	◉	4794	0.48 (0.47–0.5)	◉	897	0.46 (0.43–0.49)	◉
Hepatobiliary disorders	576	2.82 (2.6–3.07)	◉	918	0.9 (0.84–0.96)	◉	179	0.9 (0.77–1.04)	◉
General disorders and administration site conditions	3878	0.98 (0.94–1.01)	◉	27777	1.57 (1.55–1.6)	◉	2479	0.61 (0.58–0.63)	◉
Eye disorders	317	0.7 (0.63–0.78)	◉	2702	1.23 (1.18–1.28)	◉	347	0.8 (0.72–0.89)	◉
Congenital, familial and genetic disorders	305	4.48 (4–5.02)	◉	90	0.26 (0.22–0.33)	◉	130	1.97 (1.66–2.34)	◉
Ear and labyrinth disorders	42	0.42 (0.31–0.57)	◉	308	0.63 (0.57–0.71)	◉	46	0.48 (0.36–0.65)	◉
Endocrine disorders	28	0.49 (0.34–0.71)	◉	116	0.41 (0.34–0.49)	◉	19	0.34 (0.22–0.54)	◉
Immune system disorders	48	0.19 (0.14–0.25)	◉	225	0.18 (0.16–0.21)	◉	45	0.19 (0.14–0.25)	◉
Infections and infestations	1220	1.03 (0.97–1.09)	◉	5116	0.87 (0.85–0.9)	◉	1484	1.33 (1.26–1.4)	◉
Injury, poisoning and procedural complications	602	0.24 (0.22–0.26)	◉	4239	0.35 (0.34–0.36)	◉	626	0.26 (0.24–0.28)	◉
Investigations	1557	1.12 (1.07–1.18)	◉	5276	0.76 (0.74–0.78)	◉	834	0.61 (0.57–0.65)	◉
Metabolism and nutrition disorders	677	1.39 (1.28–1.5)	◉	3756	1.57 (1.52–1.63)	◉	1108	2.42 (2.28–2.57)	◉
Musculoskeletal and connective tissue disorders	386	0.32 (0.29–0.35)	◉	2337	0.39 (0.38–0.41)	◉	393	0.34 (0.3–0.37)	◉
Neoplasms benign, malignant and unspecified	3111	5.85 (5.63–6.08)	◉	6118	2.14 (2.08–2.19)	◉	2639	5.07 (4.87–5.28)	◉
Pregnancy, puerperium and perinatal conditions	5	0.05 (0.02–0.12)	◉	21	0.04 (0.03–0.07)	◉	1	0.01 (0–0.08)	◉
Product issues	11	0.03 (0.02–0.05)	◉	105	0.06 (0.05–0.07)	◉	14	0.04 (0.02–0.07)	◉
Psychiatric disorders	290	0.22 (0.19–0.24)	◉	1604	0.24 (0.23–0.25)	◉	269	0.21 (0.18–0.23)	◉
Reproductive system and breast disorders	62	0.3 (0.24–0.39)	◉	203	0.2 (0.18–0.23)	◉	61	0.31 (0.24–0.4)	◉
Skin and subcutaneous tissue disorders	1954	1.66 (1.59–1.74)	◉	15667	2.89 (2.84–2.94)	◉	2695	2.49 (2.39–2.59)	◉
Social circumstances	50	0.47 (0.36–0.63)	◉	134	0.26 (0.22–0.31)	◉	18	0.18 (0.11–0.28)	◉
Surgical and medical procedures	52	0.17 (0.13–0.22)	◉	419	0.28 (0.25–0.31)	◉	115	0.39 (0.33–0.47)	◉
Vascular disorders	278	0.57 (0.5–0.64)	◉	1769	0.74 (0.7–0.77)	◉	217	0.46 (0.4–0.53)	◉

	Dacomitinib (N = 1910)			Osimertinib (N = 41297)			Vandetanib (N = 5113)		
SOC	N	ROR (95%CI)		N	ROR (95%CI)		N	ROR (95%CI)	
Blood and lymphatic system disorders	20	0.62 (0.4–0.96)	◉	1034	1.5 (1.41–1.6)	◉	59	0.68 (0.53–0.88)	◉
Cardiac disorders	28	0.55 (0.38–0.79)	◉	1429	1.31 (1.25–1.39)	◉	113	0.83 (0.69–1)	◉
Gastrointestinal disorders	222	1.41 (1.23–1.63)	◉	3013	0.85 (0.82–0.88)	◉	722	1.77 (1.63–1.91)	◉
Renal and urinary disorders	19	0.51 (0.33–0.81)	◉	417	0.52 (0.47–0.57)	◉	121	1.24 (1.04–1.49)	◉
Respiratory, thoracic and mediastinal disorders	132	1.5 (1.26–1.79)	◉	3244	1.73 (1.67–1.79)	◉	268	1.12 (0.99–1.27)	◉
Nervous system disorders	69	0.4 (0.32–0.51)	◉	1880	0.51 (0.49–0.54)	◉	349	0.79 (0.7–0.88)	◉
Hepatobiliary disorders	31	1.78 (1.25–2.54)	◉	647	1.72 (1.59–1.86)	◉	38	0.81 (0.59–1.11)	◉
General disorders and administration site conditions	377	1.16 (1.04–1.3)	◉	13060	2.19 (2.15–2.24)	◉	637	0.67 (0.62–0.73)	◉
Eye disorders	24	0.63 (0.42–0.94)	◉	521	0.63 (0.58–0.69)	◉	117	1.16 (0.96–1.39)	◉
Congenital, familial and genetic disorders	1	0.17 (0.02–1.22)	◉	438	3.52 (3.21–3.87)	◉	7	0.45 (0.21–0.94)	◉
Ear and labyrinth disorders	9	1.08 (0.56–2.08)	◉	95	0.53 (0.43–0.64)	◉	21	0.94 (0.61–1.45)	◉
Endocrine disorders	3	0.62 (0.2–1.92)	◉	59	0.56 (0.44–0.73)	◉	20	1.55 (1–2.4)	◉
Immune system disorders	5	0.24 (0.1–0.57)	◉	124	0.27 (0.23–0.32)	◉	18	0.32 (0.2–0.51)	◉
Infections and infestations	109	1.1 (0.9–1.33)	◉	1426	0.65 (0.61–0.68)	◉	247	0.92 (0.81–1.05)	◉
Injury, poisoning and procedural complications	76	0.36 (0.29–0.46)	◉	1615	0.36 (0.34–0.37)	◉	244	0.44 (0.39–0.5)	◉
Investigations	284	2.66 (2.35–3.02)	◉	2168	0.84 (0.81–0.88)	◉	589	1.98 (1.82–2.16)	◉
Metabolism and nutrition disorders	35	0.84 (0.6–1.17)	◉	995	1.11 (1.04–1.18)	◉	168	1.53 (1.31–1.78)	◉
Musculoskeletal and connective tissue disorders	40	0.39 (0.29–0.54)	◉	1040	0.47 (0.44–0.5)	◉	222	0.83 (0.73–0.95)	◉
Neoplasms benign, malignant and unspecified	100	2.03 (1.66–2.48)	◉	4333	4.31 (4.18–4.45)	◉	125	0.92 (0.77–1.1)	◉
Pregnancy, puerperium and perinatal conditions	0		◉	2	0.01 (0–0.04)	◉	1	0.04 (0.01–0.32)	◉
Product issues	1	0.03 (0–0.23)	◉	50	0.07 (0.06–0.1)	◉	7	0.08 (0.04–0.18)	◉
Psychiatric disorders	20	0.18 (0.11–0.27)	◉	403	0.16 (0.15–0.18)	◉	118	0.39 (0.33–0.47)	◉
Reproductive system and breast disorders	5	0.29 (0.12–0.7)	◉	53	0.14 (0.11–0.19)	◉	27	0.59 (0.4–0.85)	◉
Skin and subcutaneous tissue disorders	271	2.91 (2.56–3.31)	◉	2454	1.11 (1.07–1.16)	◉	627	2.46 (2.26–2.67)	◉
Social circumstances	2	0.22 (0.06–0.9)	◉	55	0.29 (0.22–0.37)	◉	8	0.34 (0.17–0.67)	◉
Surgical and medical procedures	11	0.43 (0.24–0.77)	◉	58	0.1 (0.08–0.13)	◉	94	1.38 (1.13–1.7)	◉
Vascular disorders	16	0.39 (0.24–0.63)	◉	684	0.77 (0.71–0.83)	◉	146	1.34 (1.14–1.58)	◉

The ROR (95% CI) is followed by indicators, with red denoting positive signals and green indicating negative signals. CI: confidence interval.

#### 3.2.3 Specific AEs

Utilizing PT criteria for the analysis of specific AEs, the FAERS database has documented over 4,982 distinct types of AEs. Based on their incidence and clinical relevance, 20 AEs warrant attention: diarrhea, rash, nausea/vomiting, fatigue, decreased appetite, dyspnea, pneumonia, asthenia, dry skin, pruritus, weight loss, stomatitis, pleural effusion, pyrexia, interstitial lung disease, acne, anemia, constipation, respiratory failure, and pulmonary embolism. Among these, Gefitinib exhibited the strongest signals for dyspnea (ROR025 = 1.16), pneumonia (ROR025 = 1.87), pleural effusion (ROR025 = 7.38), interstitial lung disease (ROR025 = 18.62), and respiratory failure (ROR025 = 3.93). Erlotinib was associated with pronounced signals for rash (ROR025 = 8.38), decreased appetite (ROR025 = 3.26), dry skin (ROR025 = 4.96), anemia (ROR025 = 1.84), and pulmonary embolism (ROR025 = 2.32). Afatinib showed prominent signals for diarrhea (ROR025 = 9.17), nausea/vomiting (ROR025 = 21.03), and fatigue (ROR025 = 4.56). Dacomitinib predominantly caused stomatitis (ROR025 = 3.73). Osimertinib’s signal for interstitial lung disease (ROR025 = 13) was second only to Gefitinib and significantly higher compared to other EGFR-TKIs. Vandetanib displayed the strongest signal for acne (ROR025 = 10.56), as detailed in Table 9.

**TABLE 9:** Signal profiles of AEs induced by EGFR-TKIs at the PT level.

PT	Gefitinib (N = 22653)			Erlotinib (N = 111361)			Afatinib (N = 21758)		
	N	ROR (95%CI)		N	ROR (95%CI)		N	ROR (95%CI)	
Rash	534	3.3 (3.03–3.6)	◉	6494	8.59 (8.38–8.81)	◉	803	5.25 (4.89–5.63)	◉
Diarrhoea	711	3.14 (2.92–3.39)	◉	4808	4.4 (4.28–4.53)	◉	1956	9.61 (9.17–10.06)	◉
Nausea/Vomiting	259	0.89 (0.79–1.01)	◉	1902	1.34 (1.28–1.41)	◉	468	23.06 (21.03–25.28)	◉
Fatigue	153	0.54 (0.46–0.63)	◉	1958	1.42 (1.36–1.48)	◉	421	5.02 (4.56–5.53)	◉
Decreased appetite	252	2.86 (2.53–3.24)	◉	1477	3.43 (3.26–3.62)	◉	418	1.51 (1.38–1.67)	◉
Dyspnoea	271	1.3 (1.16–1.47)	◉	1273	1.24 (1.18–1.32)	◉	232	0.85 (0.75–0.97)	◉
Pneumonia	261	2.11 (1.87–2.38)	◉	1087	1.78 (1.68–1.89)	◉	226	1.7 (1.49–1.94)	◉
Asthenia	167	1.2 (1.03–1.4)	◉	1079	1.59 (1.5–1.69)	◉	198	1.66 (1.44–1.91)	◉
Dry skin	167	3.68 (3.16–4.28)	◉	1161	5.26 (4.96–5.57)	◉	192	1.95 (1.69–2.25)	◉
Pruritus	148	1.08 (0.92–1.28)	◉	990	1.48 (1.39–1.58)	◉	184	0.92 (0.79–1.06)	◉
Weight decreased	118	1.15 (0.96–1.37)	◉	828	1.64 (1.53–1.76)	◉	176	4.04 (3.48–4.69)	◉
Stomatitis	72	3.32 (2.63–4.18)	◉	544	5.15 (4.73–5.6)	◉	153	5.46 (4.66–6.4)	◉
Pleural effusion	193	8.5 (7.38–9.8)	◉	566	5.08 (4.68–5.52)	◉	152	6.95 (5.93–8.16)	◉
Pyrexia	217	1.7 (1.49–1.95)	◉	639	1.02 (0.94–1.1)	◉	152	1.16 (0.99–1.36)	◉
Interstitial lung disease	348	20.71 (18.62–23.04)	◉	296	3.53 (3.15–3.96)	◉	140	1.14 (0.97–1.35)	◉
Acne	88	3 (2.44–3.7)	◉	733	5.14 (4.78–5.53)	◉	134	8.18 (6.9–9.7)	◉
Anaemia	108	1.51 (1.25–1.83)	◉	693	1.98 (1.84–2.14)	◉	97	1.41 (1.16–1.73)	◉
Constipation	54	0.71 (0.54–0.92)	◉	532	1.42 (1.3–1.55)	◉	90	1.23 (1–1.51)	◉
Respiratory failure	127	4.68 (3.93–5.57)	◉	463	3.48 (3.17–3.81)	◉	73	2.79 (2.22–3.51)	◉
Pulmonary embolism	78	2.15 (1.72–2.68)	◉	453	2.54 (2.32–2.79)	◉	53	1.52 (1.16–1.99)	◉

	Dacomitinib (N = 1910)			Osimertinib (N = 41297)			Vandetanib (N = 5113)		
PT	N	ROR (95%CI)		N	ROR (95%CI)		N	ROR (95%CI)	
Rash	82	6.13 (4.92–7.65)	◉	494	1.66 (1.52–1.81)	◉	154	4.25 (3.62–4.99)	◉
Diarrhoea	78	4.13 (3.29–5.18)	◉	856	2.05 (1.92–2.2)	◉	266	5.32 (4.7–6.02)	◉
Nausea/Vomiting	10	0.41 (0.22–0.76)	◉	370	0.7 (0.63–0.77)	◉	82	1.26 (1.01–1.57)	◉
Fatigue	10	0.42 (0.22–0.78)	◉	478	0.93 (0.85–1.01)	◉	135	2.15 (1.81–2.55)	◉
Decreased appetite	21	2.83 (1.84–4.34)	◉	465	2.9 (2.65–3.18)	◉	46	2.31 (1.73–3.09)	◉
Dyspnoea	24	1.37 (0.92–2.05)	◉	402	1.06 (0.96–1.17)	◉	67	1.43 (1.12–1.82)	◉
Pneumonia	15	1.43 (0.86–2.38)	◉	280	1.23 (1.1–1.39)	◉	36	1.28 (0.92–1.78)	◉
Asthenia	18	1.54 (0.97–2.45)	◉	279	1.1 (0.98–1.24)	◉	50	1.6 (1.21–2.12)	◉
Dry skin	12	3.13 (1.77–5.52)	◉	201	2.42 (2.11–2.78)	◉	35	3.41 (2.45–4.76)	◉
Pruritus	25	2.19 (1.47–3.25)	◉	162	0.65 (0.56–0.76)	◉	34	1.1 (0.79–1.55)	◉
Weight decreased	16	1.85 (1.13–3.02)	◉	246	1.31 (1.16–1.49)	◉	23	0.99 (0.66–1.49)	◉
Stomatitis	12	6.58 (3.73–11.6)	◉	170	4.31 (3.71–5.01)	◉	18	3.67 (2.31–5.84)	◉
Pleural effusion	13	6.76 (3.92–11.66)	◉	327	7.91 (7.09–8.83)	◉	6	1.16 (0.52–2.58)	◉
Pyrexia	15	1.39 (0.84–2.32)	◉	215	0.92 (0.81–1.05)	◉	24	0.83 (0.56–1.24)	◉
Interstitial lung disease	4	2.76 (1.04–7.37)	◉	439	14.29 (13–15.71)	◉	4	1.03 (0.39–2.75)	◉
Acne	5	2.02 (0.84–4.86)	◉	49	0.91 (0.69–1.21)	◉	86	13.18 (10.65–16.31)	◉
Anaemia	3	0.5 (0.16–1.54)	◉	144	1.11 (0.94–1.3)	◉	8	0.5 (0.25–0.99)	◉
Constipation	5	0.78 (0.32–1.87)	◉	117	0.84 (0.7–1.01)	◉	24	1.39 (0.93–2.08)	◉
Respiratory failure	5	2.17 (0.9–5.23)	◉	141	2.84 (2.41–3.35)	◉	6	0.97 (0.44–2.17)	◉
Pulmonary embolism	8	2.61 (1.3–5.23)	◉	164	2.48 (2.13–2.89)	◉	13	1.58 (0.92–2.73)	◉

The ROR (95% CI) is followed by indicators, with red denoting positive signals and green indicating negative signals.

#### 3.2.4 Onset time of AEs

**FIGURE 8:**
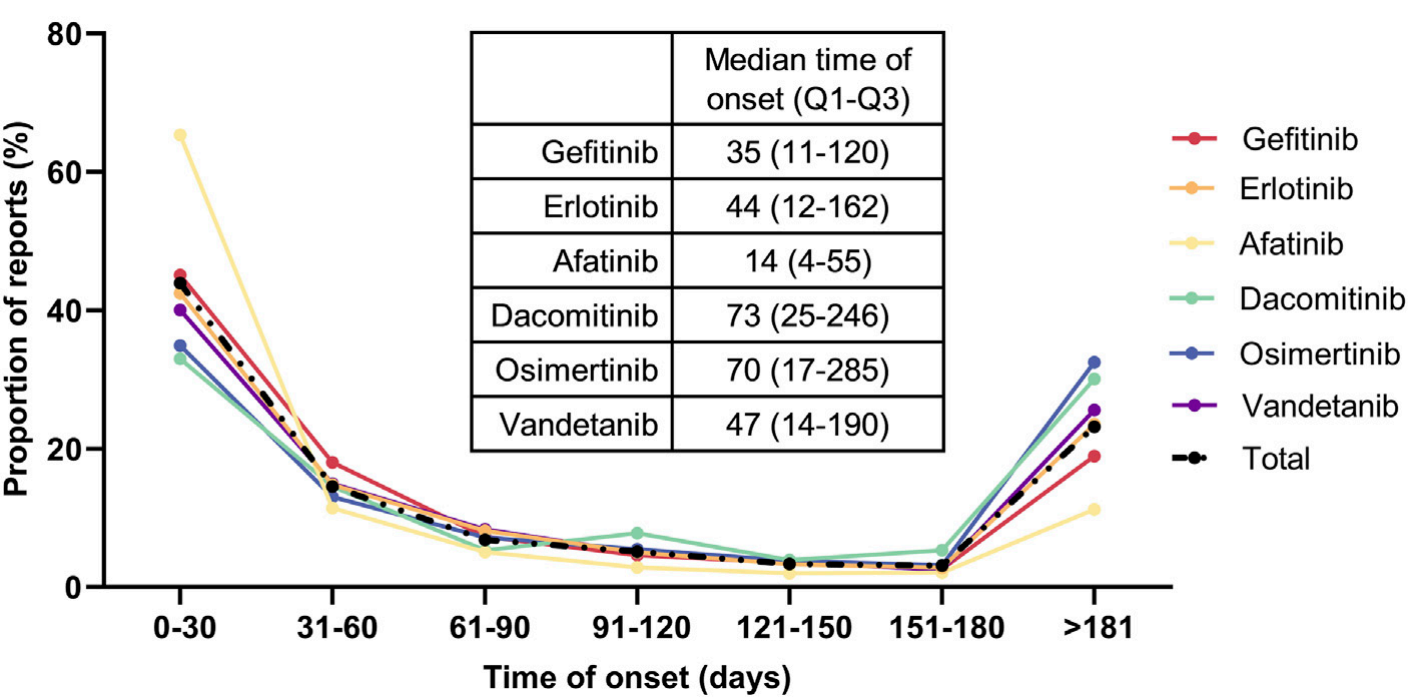
Time to onset of AEs Induced by different EGFR-TKIs.

Figure 8 illustrates the time to onset of AEs following the initiation of EGFR-TKI therapy, along with their median and interquartile range (IQR). Following the commencement of EGFR-TKI treatment, 43.9% of AEs occurred within 30 days, with Afatinib exhibiting the highest proportion at 65.3%. An additional 14.6% of AEs transpired between 31 and 60 days post-treatment start. Moreover, 23.1% of AEs emerged more than 181 days after initiating therapy, with Osimertinib accounting for the highest percentage at 32.5%. The shortest median time to AE onset was observed with Afatinib, at 14 days (IQR: 4–55 days), while Dacomitinib had the longest median time at 73 days (IQR: 25–246 days), followed by Osimertinib (median: 70 days, IQR: 17–285 days). The median times to AE onset for other drugs ranged from 35 to 47 days.

**FIGURE 9:**
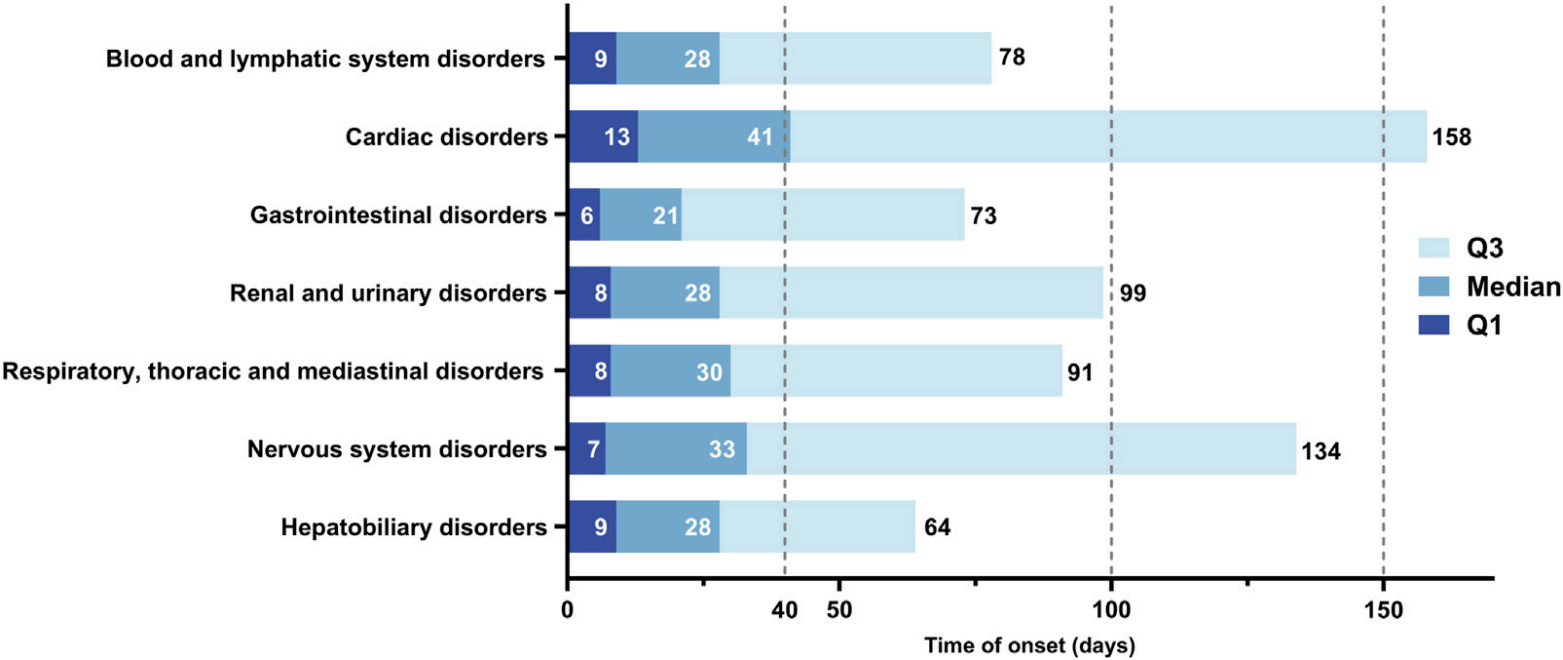
Time to onset of AEs in different systems induced by EGFR-TKIs.

We conducted a statistical analysis on the median occurrence time and interquartile ranges of systemic AEs associated with EGFR-TKIs, categorized by SOC (Figure 9). Most systemic AEs occurred within 30 days, except for Cardiac Disorders, which had a median onset time of 41 days. The second longest median onset time was observed in Nervous System Disorders at 33 days, while Gastrointestinal Disorders had the shortest median onset time at 21 days. Notably, the third quartile (Q3) for the onset time of Cardiac Disorders was 158 days, followed by Nervous System Disorders at 134 days; the Q3 for all other AEs was less than 100 days.

#### 3.2.5 Fatality rate of AEs

**FIGURE 10:**
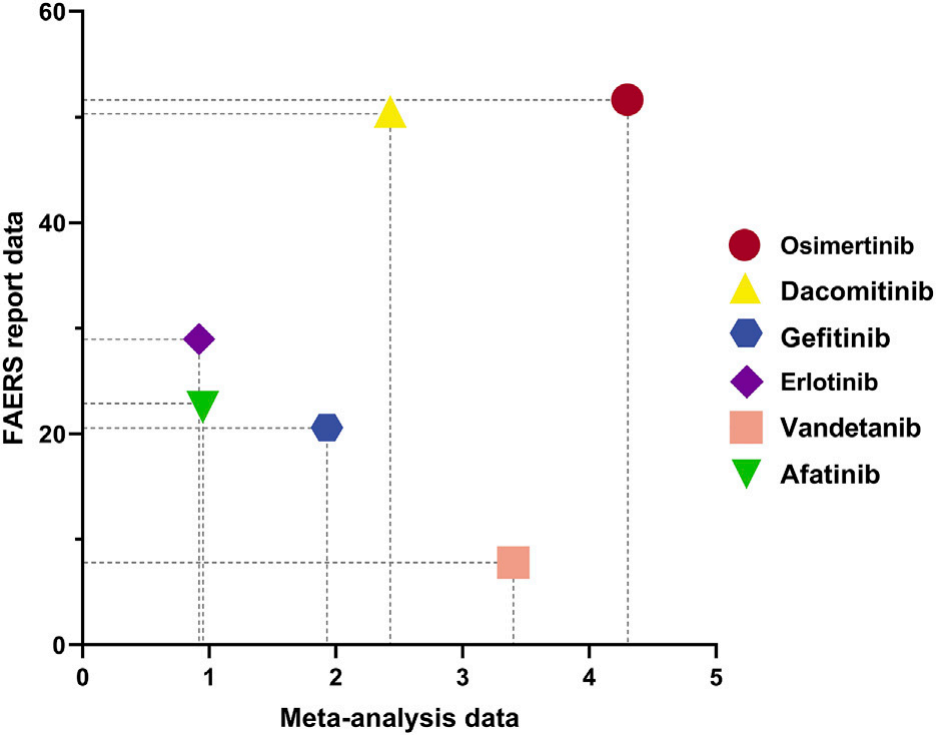
Mortality of AEs induced by different EGFR-TKIs in two research methods.

Figure 10 illustrates the mortality rates of various drugs as determined by different research methodologies. The y-axis represents the proportion of deaths following AEs associated with different EGFR-TKIs in the FAERS database, ranked from highest to lowest mortality rate as follows: Osimertinib (51.66%), Dacomitinib (50.53%), Erlotinib (28.98%), Afatinib (22.53%), Gefitinib (20.6%), and Vandetanib (7.81%). The x-axis shows the pooled proportions of deaths due to AEs for different EGFR-TKIs as summarized by a NMA, with rankings from highest to lowest as Osimertinib (4.3%), Vandetanib (3.4%), Dacomitinib (2.43%), Gefitinib (1.93%), Afatinib (0.95%), and Erlotinib (0.92%). Based on this data, we conducted an exploratory study attempting to multiply the AEs mortality rates of the aforementioned EGFR-TKIs across two research settings (representing real-world data through DA and clinical trial environments via NMA). In Figure 10, this is depicted as the area of the rectangle formed by each drug’s point and the origin, with the resulting product of mortality rates used to rank the EGFR-TKIs as follows: Osimertinib, Dacomitinib, Gefitinib, Erlotinib, Vandetanib, and Afatinib (Table 10).

**TABLE 10:** Mortality rates of AEs induced by different EGFR-TKIs in two research methods.

	FAERS analysis data			Meta-analysis data			AE fatality rate product area
	No. of deaths	N	Fatality Rate(%)	No. of deaths	N	Fatality Rate(%)	
Osimertinib	10385	20103	51.66	12	279	4.3	222.14
Dacomitinib	285	564	50.53	17	699	2.43	122.79
Gefitinib	1480	7184	20.6	62	3217	1.93	39.76
Erlotinib	11639	40159	28.98	27	2934	0.92	26.66
Vandetanib	105	1344	7.81	24	706	3.4	26.55
Afatinib	1316	5842	22.53	15	1571	0.95	21.4

The AE fatality rate product area is the product of the percentage mortality rate of AEs for a specific EGFR-TKI in a network meta-analysis and the percentage mortality rate of AEs in the FAERS database.

**FIGURE 11:**
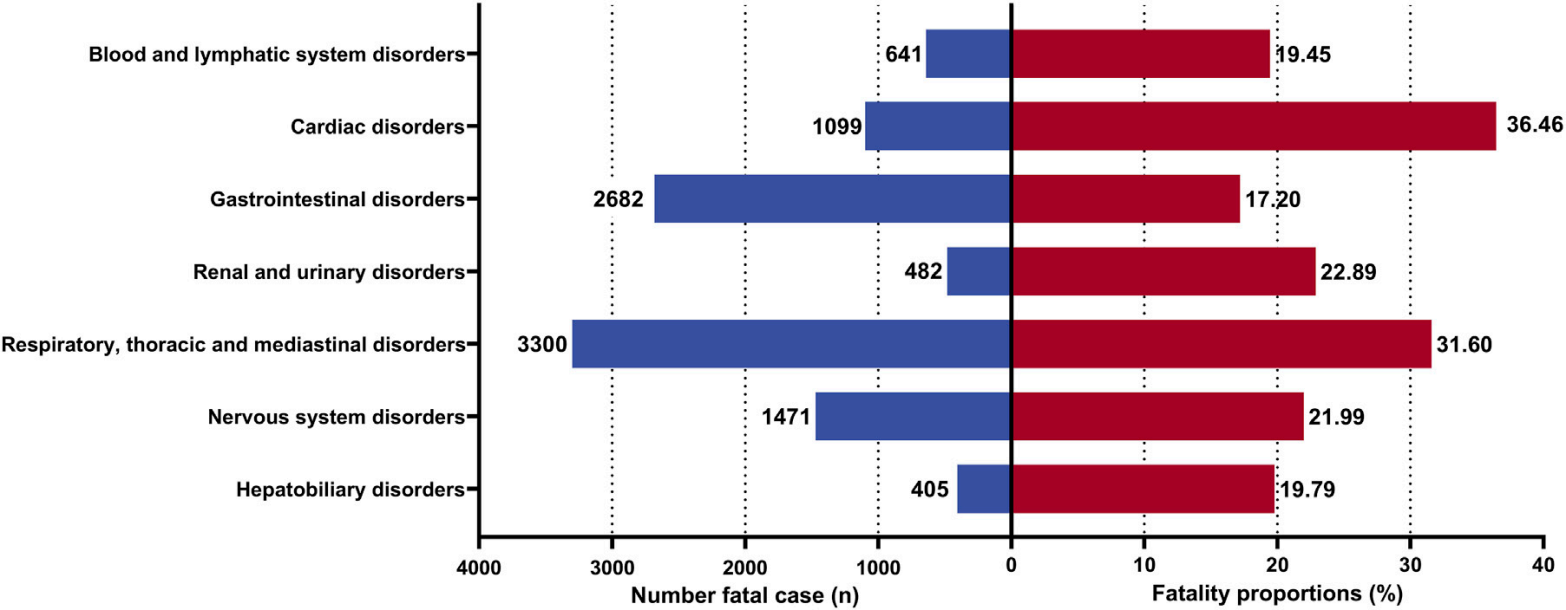
Mortality of AEs in different systems induced by EGFR-TKIs.

Finally, we analyzed the data from the FAERS database to determine the number of death cases and the mortality rates of AEs across different systems. The highest mortality rate was observed in Cardiac disorders at 36.46% (n = 1099), followed by Respiratory, thoracic, and mediastinal disorders at 31.6% (n = 3300). Subsequently, the mortality rates for other AEs ranked from highest to lowest were: Renal and urinary disorders (22.89%, n = 482), Nervous system disorders (21.99%, n = 1471), Hepatobiliary disorders (19.79%, n = 405), Blood and lymphatic system disorders (19.45%, n = 641), and Gastrointestinal disorders (17.2%, n = 2682) (Figure 11).

## 4 Discussion

To our knowledge, this study represents the inaugural endeavor to characterize and analyze AEs associated with EGFR-TKIs by integrating two distinct methodologies. Specifically, an NMA was conducted based on RCTs comparing EGFR-TKIs either against each other or versus chemotherapy, deliberately excluding studies involving combination therapy with EGFR-TKIs to minimize confounding effects from additional medications. Trials incorporating placebo controls were also omitted due to their tendency to enroll healthier patient populations, potentially diverging from real-world scenarios. Concurrently, a DA was performed utilizing the FAERS database, a repository established to facilitate the FDA’s post-market surveillance of drugs and therapeutic biologics, which encompasses comprehensive and standardized reports of all AEs collected by the FDA. By merging these approaches, the FAERS dataset furnished expansive real-world evidence, while RCTs contributed high-quality experimental data, thereby facilitating a more holistic and precise assessment of EGFR-TKI safety.

Our NMA revealed several key findings. First, over 80% of EGFR-TKI users experienced AEs, although the incidence of high-grade AEs (≥3) was relatively low at 17.7%. However, the rates for Osimertinib (37.8%) and Dacomitinib (41.6%) were significantly higher, suggesting that these drugs may require more cautious use in specific patient populations. Furthermore, DA showed that Osimertinib was not only significantly associated with Blood and lymphatic system disorders, Gastrointestinal disorders, and Renal and urinary disorders but was also the only EGFR-TKI to yield a positive signal for Cardiac disorders. This finding is particularly important for patients with a history of cardiac diseases or impaired cardiac function, as cardiovascular AEs such as QT prolongation, reduced left ventricular ejection fraction (LVEF), and heart failure can severely impact their quality of life and prognosis. Therefore, when prescribing Osimertinib, physicians should closely monitor electrocardiograms and cardiac function indicators to promptly identify and manage potential cardiovascular risks. In contrast, Dacomitinib was more frequently associated with Respiratory, thoracic and mediastinal disorders, Nervous system disorders, and Hepatobiliary disorders. These AEs can also negatively affect patients’ health and quality of life. Hence, when using Dacomitinib, physicians should also monitor the respiratory, nervous, and hepatobiliary systems and tailor treatment plans based on the patient’s specific conditions and potential risks.

We further employed two methods to delve into the specific AEs associated with different EGFR-TKIs. On one hand, we calculated RORs based on FAERS data; on the other, we aggregated data from randomized controlled trials to assess the incidence rates of specific AEs induced by various EGFR-TKIs. In both research approaches, rankings were derived from respective datasets, revealing an overlap of ten out of the top twenty AEs, encompassing Rash, Nausea/Vomiting, Fatigue, Dyspnea, Pneumonia, Dry Skin, Stomatitis, Anorexia, Interstitial Lung Disease (ILD), and Anemia. Among these prevalent and critically concerning AEs, some like Rash, Nausea/Vomiting, Fatigue, Dry Skin, Stomatitis, and Anorexia may ameliorate through dose adjustment or symptomatic treatment. Conversely, others such as Dyspnea, Pneumonia, ILD, and Anemia could necessitate treatment interruption or cessation, severely impacting prognosis or causing irreversible physiological alterations, including unintended mortality, particularly warranting vigilance towards respiratory complaints and diseases. Beyond common AEs, META analysis highlighted additional top twenty AEs including Alopecia, Constipation, Myalgia/Arthralgia, Elevated AST, Elevated ALT, Increased Creatinine Levels, Leukopenia, Thrombocytopenia, Insomnia, and Chest Pain. Meanwhile, FAERS data underscored ROR-prominent AEs comprising Diarrhea, Asthenia, Pruritus, Weight Loss, Pleural Effusion, Pyrexia, Acne, Constipation, Respiratory Failure, and Pulmonary Embolism. This discrepancy suggests that clinical trial reports tend to emphasize laboratory test abnormalities, whereas physician- or patient-reported outcomes lean towards subjective experiences. It underscores the necessity not only to prioritize these AEs to prevent potential severe consequences but also to intensify laboratory monitoring during EGFR-TKI therapy to ensure timely detection of AEs, thereby mitigating diagnostic omissions and associated risks.

Research based on the FAERS database has revealed the temporal distribution characteristics of AEs during EGFR-TKI treatment. The study found that most AEs occur within 60 days of treatment initiation, with no significant differences observed among various EGFR-TKIs. However, within the first 30 days of treatment, Afatinib had the highest proportion of AEs, while after 180 days of treatment, Osimertinib exhibited the highest proportion. Further analysis showed that gastrointestinal disorders had the shortest median onset time at 21 days, whereas cardiac disorders had the longest median onset time at 41 days. Additionally, the ROR for gastrointestinal disorders caused by Afatinib was significantly higher than that for other EGFR-TKIs, and the ROR for cardiac disorders caused by Osimertinib was markedly higher than that for other EGFR-TKIs. These findings indicate that Afatinib is associated with a higher incidence of gastrointestinal AEs, which generally occur early in the treatment course. Conversely, Osimertinib may lead to a higher incidence of cardiac disorders, which usually occur later in the treatment process. This discovery underscores the importance of carefully considering the risk of cardiac disorders when selecting an EGFR-TKI for clinical practice, especially during long-term treatment.

Research based on the FAERS database has revealed the temporal distribution patterns of AEs during EGFR-TKI therapy. It was found that most AEs occur within the first 60 days of treatment, with no significant differences observed among various EGFR-TKIs. However, Afatinib had the highest proportion of AEs occurring within the first 30 days of treatment, whereas Osimertinib had the highest proportion of AEs after 180 days of treatment. Further analysis showed that gastrointestinal disorders had the shortest median time to occurrence at 21 days, while cardiac disorders had the longest at 41 days. Additionally, Afatinib-induced gastrointestinal disorders had a significantly higher ROR025 than other EGFR-TKIs, and Osimertinib-induced cardiac disorders had a notably higher ROR025 compared to other EGFR-TKIs. This suggests that Afatinib is associated with a higher incidence of gastrointestinal AEs, which typically occur early in the treatment period. Conversely, Osimertinib may be associated with a higher incidence of cardiac disorders, which tend to occur later. This finding underscores the need for careful consideration of cardiac risk, particularly during long-term treatment, when selecting an EGFR-TKI in clinical practice.

We conducted an exploratory study on the mortality rate associated with AEs. First, discrepancies in mortality rates between the FAERS database and RCTs included in the META-analysis primarily arise from differences in data collection methods. The FAERS database relies on spontaneous reporting, which may include more complex and severe cases, leading to higher mortality rates. In contrast, RCTs are conducted under stringent conditions with a relatively homogeneous patient population. Additionally, variations in patient demographics, medication usage, and statistical methodologies could further influence the results. Second, an analysis of death cases due to AEs across different systems within the FAERS database revealed that respiratory system-related AEs had the highest number of deaths, while cardiovascular AEs had the highest mortality rate. Respiratory issues may be linked to drug-induced damage to lung cells, whereas cardiovascular events, once occurred, have a high mortality rate possibly due to interference with cardiac cell function. Third, we combined data from the FAERS database and RCTs for the first time to compare the mortality rates of different EGFR-TKI-related AEs. Although the statistical interpretation might be limited, the multiplicative results were consistent with expectations, indicating that both datasets reflect similar drug risks. Notably, Osimertinib was associated with the highest mortality rate among EGFR-TKIs, especially for cardiovascular-related AEs. This suggests potential cardiovascular safety concerns with Osimertinib and corroborates previous findings about its association with delayed cardiac AEs. Therefore, it is imperative to enhance long-term monitoring and follow-up of patients treated with Osimertinib to promptly detect and manage cardiovascular issues, thereby preventing patient mortality due to cardiovascular AEs.

## 5 Limitations

This study, despite employing a variety of analytical methods, has certain limitations. Firstly, the spontaneous reporting nature of FAERS data may introduce bias into the results. Secondly, NMA is constrained by the quality and heterogeneity of the included studies, which may potentially affect the accuracy of the outcomes. Additionally, factors such as heterogeneity within patient populations and insufficient consideration of individual differences may impact the generalizability and comprehensiveness of the findings.

## 6 Conclusion

This study employed a comprehensive approach combining NMA and DA from the FAERS database to examine AEs associated with EGFR-TKIs. The results indicated that different EGFR-TKIs are associated with distinct AE profiles, predominantly characterized by relatively mild events such as Rash and Nausea. However, Osimertinib and Dacomitinib exhibited higher rates of high-grade AEs, with Osimertinib showing a significant association with cardiac disease risk. Additionally, AEs were frequently observed at the onset of treatment, but Osimertinib was found to cause more delayed AEs and had the highest mortality rate among these events. Therefore, when prescribing EGFR-TKIs, physicians should thoroughly assess patient conditions and closely monitor for AEs, especially cardiac function, regularly, to ensure patient safety.

## Data Availability

The original contributions presented in the study are included in the article/supplementary material, further inquiries can be directed to the corresponding authors.
